# Integrated Actuation and Sensing: Toward Intelligent Soft Robots

**DOI:** 10.34133/cbsystems.0105

**Published:** 2024-04-18

**Authors:** Shuai Zhou, Yuanhang Li, Qianqian Wang, Zhiyang Lyu

**Affiliations:** Jiangsu Key Laboratory for Design and Manufacture of Micro-Nano Biomedical Instruments, School of Mechanical Engineering, Southeast University, Nanjing 211189, China.

## Abstract

Soft robotics has received substantial attention due to its remarkable deformability, making it well-suited for a wide range of applications in complex environments, such as medicine, rescue operations, and exploration. Within this domain, the interaction of actuation and sensing is of utmost importance for controlling the movements and functions of soft robots. Nonetheless, current research predominantly focuses on isolated actuation and sensing capabilities, often neglecting the critical integration of these 2 domains to achieve intelligent functionality. In this review, we present a comprehensive survey of fundamental actuation strategies and multimodal actuation while also delving into advancements in proprioceptive and haptic sensing and their fusion. We emphasize the importance of integrating actuation and sensing in soft robotics, presenting 3 integration methodologies, namely, sensor surface integration, sensor internal integration, and closed-loop system integration based on sensor feedback. Furthermore, we highlight the challenges in the field and suggest compelling directions for future research. Through this comprehensive synthesis, we aim to stimulate further curiosity among researchers and contribute to the development of genuinely intelligent soft robots.

## Introduction

Soft robotics is an emerging field that has garnered widespread interest and research due to its flexibility and adaptability. Soft robots offer a higher degree of mechanical variability and plasticity compared to traditional rigid robots and are capable of mimicking the properties of biological tissues and organisms [1]. This adaptability and flexibility make soft robots highly suitable for various tasks in complex environments, such as medicine, rescue, and exploration [2–4]. Unlike rigid robots, which are constructed from inflexible materials, the bodies of soft robots are made of soft and expandable materials like hydrogels that have a low modulus of elasticity, enabling them to deform and absorb much of the energy generated by collisions [5,6]. The complex structures of soft robots can be fabricated by layer-by-layer stacking materials through 3-dimensional (3D) or 4D printing technology with great manufacturing flexibility [7–9], which also allows the implementation of multiple materials in a single structure [10]. More importantly, soft robots employ a flexible muscle-like actuation technology that provides them with greater degrees of freedom compared to rigid robots [11,12]. This flexibility is further enhanced by stretchable sensing technology, which enables soft robots to sense, acquire information about their environment, and achieve intelligence and autonomy [13]. In recent years, various actuation and sensing strategies have been continuously explored by attempting diverse manufacturing and modeling methods [7,14–16], thereby extending applications of soft robots such as bionics [17–20] and intelligent equipment [21–24].

Actuation technology enables soft robots to accomplish motion and interact with the environment [25], while sensing technology allows them to perceive and acquire information about their surroundings [26]. The interaction between actuation and sensing technologies forms the core functionality of soft robots [27]. Pressure-driven [28], electrically driven [29], and specialized material like shape memory materials (SMMs) driven strategies [30] endow soft robots with the potential for complex movements such as rotation, rolling, crawling, and bending [31]. Simultaneously, sensing strategies based on electrical [32], magnetic [33,34], and specialized material responses [35] confer upon soft robots self-perceptive and adaptive characteristics, enabling them to explore the external environment and perceive changes in their form [36,37]. These elements are indispensable for the realization of intelligent soft robots. However, relying solely on actuation and sensing technologies is insufficient to create truly intelligent soft robots. Previous research has often developed actuation and sensing technologies separately, resulting in limitations in the behavior and sensing capabilities of soft robots [11,38]. To overcome these limitations, integrated actuation-sensing technology has emerged as a new research direction in recent years [39]. This technology aims to integrate actuation and sensing functions to achieve intelligence and autonomy in soft robots. Therefore, soft robots can better understand and adapt to their environment, resulting in more precise, flexible, and intelligent movements and behaviors. This integration enables soft robots to excel in various tasks and environments, boosting their overall performance and adaptability.

While extensive work has been done on soft robotics [40–45], little focus has been placed on the integration of actuation and sensing for soft robots. This review aims to explore existing popular actuation and sensing integration technologies. We will first have a systematic review of strategies for soft actuation and soft sensing in soft robots and list the advantages and limitations of various actuation and sensing strategies. We then place more discussions on strategies for integrating actuation and sensing, for example, flexible electronic skin (e-skin) for sensor surface integration and sensor internal integration. We believe that this comprehensive review will promote the development of intelligent soft robotics and provide insights into technological breakthroughs and developments in this area.

**Fig. 1.**
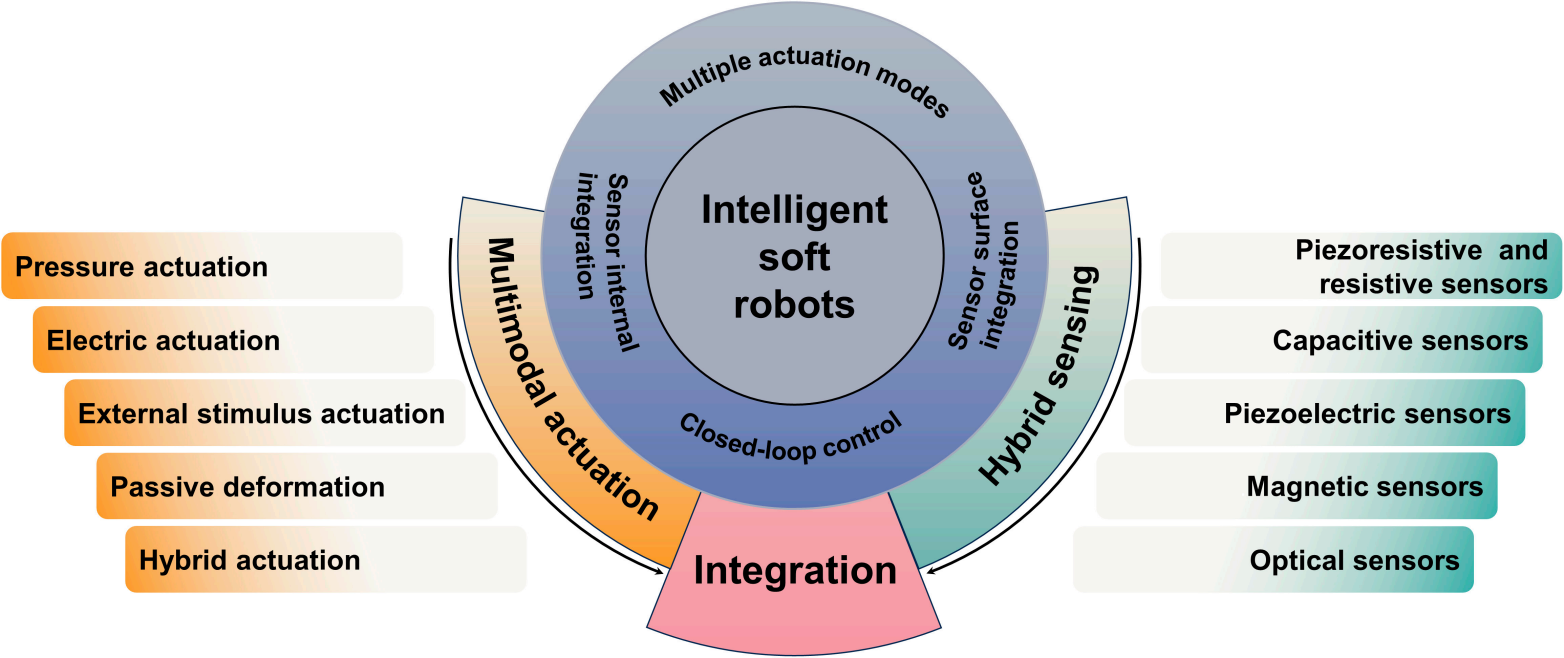
Overview of this review.

As shown in Fig. 1, this review will be structured as follows. The “Strategies for Soft Actuation” section will delve into intelligent actuation strategies for soft robots and summarize work related to multimodal actuation techniques. In the “Strategies for Soft Sensing” section, different flexible sensing strategies for soft robots, as well as the integration of proprioceptive and external sensing, will be discussed. The “Integrated Actuation and Sensing” section will provide a summary of the existing strategies for actuation-sensing integration and suggest potential areas for further exploration. Finally, the future of further integration of actuation and sensing to achieve intelligence in soft robots will be presented and concluded.

## Strategies for Soft Actuation

**Fig. 2.**
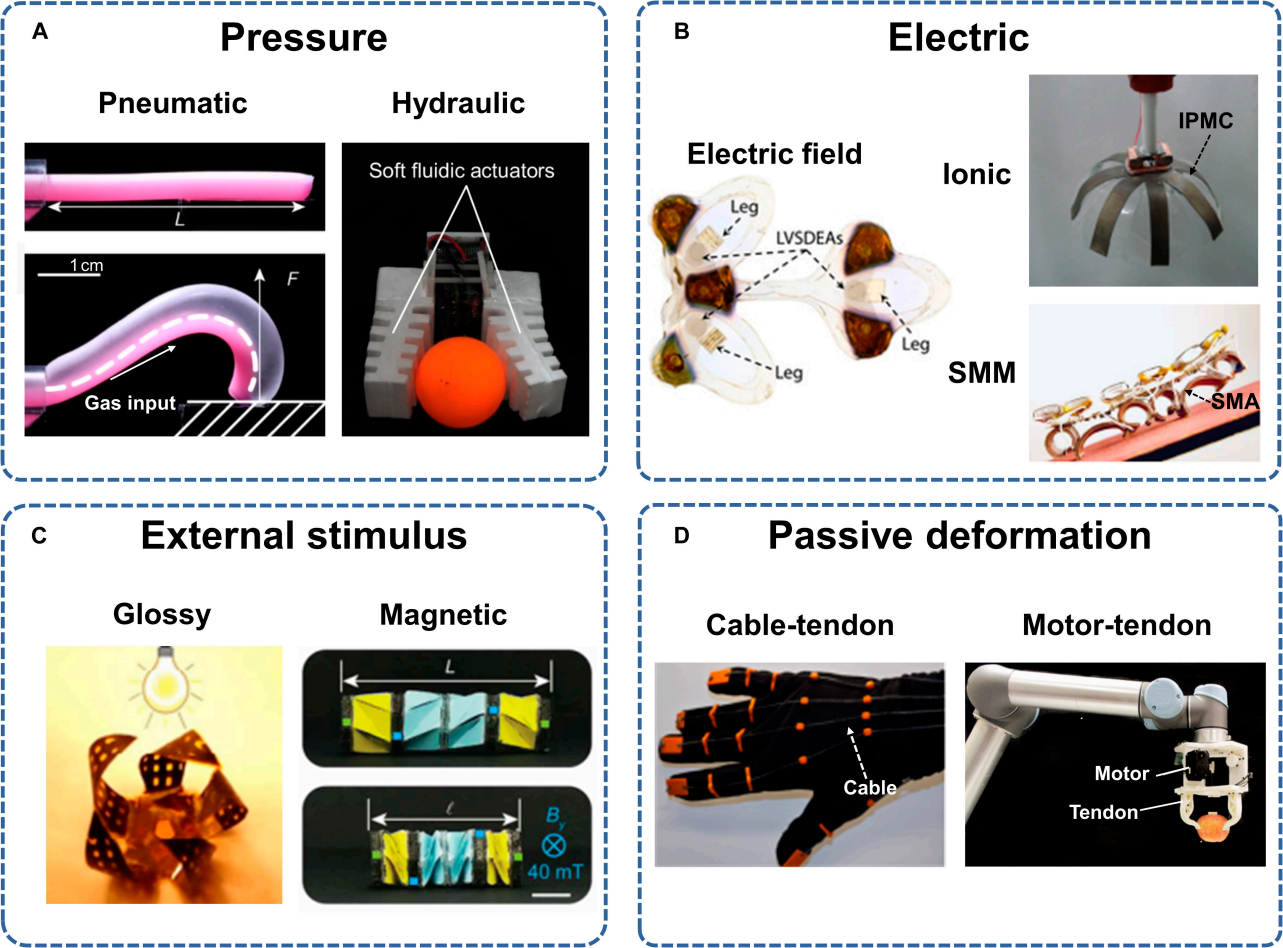
Soft actuation strategies. (A) Pressure actuation, including pneumatic and hydraulic pressure methods. Reproduced with permissions from [50,52]. (B) Electrically actuation, including dielectric elastomers (DE), ionic polymer–metal composites (IPMC), and shape memory alloy (SMA). Reproduced with permissions from [57,64,67]. (C) External stimulus actuation, including glossy and magnetic response methods. Reproduced with permissions from [72,79]. (D) Actuation based on passive deformation, including cable and servo motor actuation. Reproduced with permissions from [81,84].

Actuation strategies form a central pillar in the field of soft robotics, given the fact that they control the motion and function of these agile robots. Various actuation strategies have been explored to empower soft robots with the ability to interact with their environment and perform complex tasks. This breakdown analyzes 4 primary actuation strategies used in the field: pressure, electrical, external stimulation, and passive deformation. As shown in Fig. 2, we will examine the principles, advantages, limitations, and recent advancements associated with each strategy. We will also investigate the emerging technique of multimodal actuation, which amalgamates different actuation approaches to bolster the versatility and performance of soft robots. By delving into these diverse actuation approaches, we aim to shed light on the progress and challenges in this crucial aspect of soft robotics and pave the way for the integration of intelligent drive systems in future generations of soft robots.

### Comparison of different actuation strategies

#### Pressure actuation

Pressure actuation, a widely used soft-body actuation strategy, will be our first point of analysis. This strategy emulates the movement of some mollusks by embedding different types of fluid channels, such as cylindrical or pleated, into the elastic materials or by adding a restrictive layer of material at specific locations. Once the fluid is introduced, the various components of the mollusk analog are deformed due to changes in channel pressure, inducing contraction, expansion, and bending of the foundational movements of a mollusk. There are 2 primary types of pressure actuation strategy: pneumatic and hydraulic actuation.

*Pneumatic actuation:* Pneumatic actuation, particularly through soft pneumatic actuators (SPAs), is a prevalent technique in soft robotics. These actuators comprise an elastic body with multiple embedded void channels. Upon the application of fluid pressure, each channel undergoes expansive deformation, leading to the contraction or expansion of the internal cavities within the soft robotic system. This mechanism enables the desired objectives of deformation and motion to be achieved. Noteworthy advantages of SPA include light media mass, widespread source, minimal pollution, and satisfied-reliability requirements for soft robots. Despite offering high energy density and high flexibility, challenges arise due to their considerable mass and high sealing requirements, particularly impeding the device miniaturization. An illustrative example within the SPA domain is the pneumatic artificial muscle (PAM), where the McKibben actuator represents a quintessential task [46,47]. PAMs typically store the energy required for actuation mechanically (pressure–volume energy), contracting when pressurized. These actuators consist of a flexible or stretchable cylindrical membrane, bound by highly tensile fibril and sheltered by 2 rigid end fittings [40]. In a notable advancement, De Pascali et al. designed a novel PAM structure, where the geometry of the membrane inherently enabled the coupling of expansion and axial contraction without the need for anti-stretch components, end caps, or restraints. This innovative approach enhances versatility and scalability across a wide range of sizes and large changes in drive strength [48].

Nevertheless, a flexible soft robotic gripper based on the SPA was designed by Zhang et al. [49], which showcased successful application, in this case, gripping fragile items by inducing a change in pulse signal. Figure 2A illustrates an SPA crafted through bubble casting [50]. The gradual solidification of the elastomer allows for easy customization of its form, catering to a diverse range of applications from artificial muscles to fixtures. Through this casting-based SPA, there is an augmentation in the flexibility and expansiveness of the actuator. Utilizing the above designs, versatile SPAs can meet the shift toward preparing multifunctional soft robots that combine various technologies, mechanisms, and devices. Nonetheless, miniaturizing SPAs, which usually require numerous pneumatic fittings such as air tubes and solenoid reversing valves, presents a future research direction.

*Hydraulic actuation:* Hydraulic-driven actuation has been widely used in a range of soft robot configurations since the birth of soft robots in the 20th century. Fluids demonstrate excellent potential for soft robot drives, given their superior incompressibility, high response frequency, and minimal losses in the absence of leaks. Common hydraulic actuators are fluid-elastic and consist of dual soft elastomeric layers divided by a non-stretchable strain-limiting layer. As the fluid pressure inside the embedded channel ramps up, the channel expands, thereby bending the actuator. Owing to the robust nonlinearity and intricate geometric configuration inherent in hydraulic actuators, achieving precision of motion presents a formidable challenge. Finite element analysis is frequently employed to address nonlinear issues, forecast drive performance, and enhance the precision of hydraulic propulsion [51]. Notably, hydraulic actuation technology has recently advanced significantly in areas such as self-repair, miniaturization, and multimodal motion. For instance, Fig. 2A depicts a fluidic actuator that integrates an internal soft electro-hydraulic fluid pump for cordless propulsion, concurrently incorporating self-healing fluid to achieve automatic recovery from material damage [52]. Furthermore, Xie et al. proposed a hydraulic soft actuator for multi-degree-of-freedom spatial motion, where the cylinder soft actuator made of elastomer had 3 symmetrically distributed fiber-reinforced hydraulic chambers. By controlling the pressure vector within the chambers, the soft actuator allowed for axial elongation and bending movement [53]. In addition, Kurumaya et al. [54] integrated a hydraulically driven bellows-type flexible gripper and a fiber-reinforced flexible winding actuator into an underwater rigid robotic arm for the collection of vulnerable organisms underwater, which successfully collected coral samples non-destructively in shallow waters. In general, hydraulic actuation offers high responsiveness and drive forces, although it presents considerable challenges in modeling and control.

#### Electric actuation

The electric actuation strategy revolves around the utilization of electroactive substances, particularly focusing on their unique ability to undergo structural changes in response to applied electric fields. This leads to a diverse range of transformations, including stretching, bending, and swelling. Electrodynamic strategies within this field can be broadly classified into 3 main branches based on the difference in the underlying energy mechanism: those driven by electric fields, primarily employing dielectric elastomers (DEs), those driven by ions, prominently featuring ionic polymer–metal composites (IPMCs), and those driven by SMMs.

*DE-based actuation:* The actuation principle of DEs is mainly based on the action of an applied electric field. Under the action of the electric field, the charges on both sides of DEs are attracted to each other, thereby generating Maxwell stress. This electrostatic force causes DEs to expand in the face direction and contract in the thickness direction [29]. Due to their fast response and high energy density, DEs are currently widely used in ground robots, aerial robots, and robotic grippers. For instance, in the realm of DE-based actuators, Zhao et al. have innovatively explored methods to achieve lightweight, low-profile DE-based actuators capable of high force and substantial strain. Their approach involved layered dielectric materials and electrodes, followed by precise winding, transforming a film structure of tens of microns into a millimeter device. This transformation effectively converted the shrinkage along the thickness axis and expansion within the plane into axial elongation, leading to reduced voltage requirements and operation at lower voltage thresholds [55]. However, it is important to note that the lifetime of DE-based actuators presents a marked challenge, as exceedingly high voltages can lead to electrode breakdown. To address this issue, Jiang et al. [56] proposed a solution involving a thin mesh of carbon nanotubes (CNTs), which both enhances the self-cleaning of DEs and introduces a non-dimensional measure called capacitance retention for real-time monitoring of the drive performance during the device’s lifetime.

In addition, the persistent challenge of elevated voltage in DEs has been addressed by Ji et al. They introduced low-voltage stacked DE-based actuators, operating at voltages below 450 V, as illustrated in Fig. 2B. These actuators have been utilized to propel diminutive, cordless, and autonomous-legged robots of insect scale [57]. Their works demonstrate a high level of agreement between the actual change in driving capacity and the theoretical change, offering a promising option for ongoing assessment of DE performance. Furthermore, DEs are often used in the manufacture of flexible artificial muscles due to their high power-density properties. Chen et al. [58] explored the use of multilayer DE-based actuators to create a flexible artificial muscle with impressive specifications, achieving a resonant frequency of 500 Hz and a power density of 600 W/kg. Meanwhile, DEs are miniaturized and easy to integrate, which gives them the potential for detection in extreme environments. Li et al. [18] deployed DE artificial muscles to deliver a self-powered soft robotic fish, which was taken into the Marianas Trench at around 10,000 m. Given the extreme conditions of the deep sea, which include low temperatures and extremely high pressures, the researchers optimized the DE material to enhance the robot’s robustness and ensure that the robot’s drive performance is maintained at a respectable level over time.

Overall, DE-based actuators present several distinct advantages, like high power density, broad bandwidth, the ability to achieve remarkable strain, and impressive overall efficiency. These actuators can reliably generate strains of up to 100% and operate at frequencies ranging from hundreds to thousands of hertz [59,60]. Nevertheless, DE-based actuators have certain drawbacks, including the requirement for pre-stress, challenges in creating flexible and robust electrodes, and the use of high voltages during operation. Ongoing research endeavors are dedicated to overcoming these obstacles and advancing the field further.

*IPMC-based actuation:* IPMC has emerged as a promising material for soft actuators in electrically driven soft robotics. As a smart material, IPMC demonstrates remarkable electromechanical properties that position it as an ideal candidate for actuation within the domain of soft robotics. IPMC consists of a thin ionic polymer film sandwiched between 2 metal electrodes, usually made of platinum or gold, and exhibits unique behavior when subjected to an electric field [61]. This electrical stimulation causes the cations within the polymer to migrate toward the negative electrode, resulting in a bending or deformation response in the material. The unique properties of IPMC make it an excellent choice for soft actuation. It exhibits large bending displacements in response to low applied voltages, enabling precise and responsive control of movement. This feature is particularly advantageous in applications that require delicate manipulation or fine motor skills (FMSs). Additionally, IPMC offers a high power-to-weight ratio, facilitating efficient actuation while maintaining a relatively low overall weight of the robot [62]. For example, Chung et al. [63] demonstrated an IPMC brake prepared using nano-silver powder, which exhibited substantial deformation in bending angle and operated at a low driving voltage. IPMC has also found application in the construction and activation of bionic jellyfish robots (Fig. 2B), successfully emulating the undulating form of actual jellyfish [64]. Ongoing research continues to explore the full potential of IPMC in the field of materials.

Overall, the utilization of IPMC in soft robotics for electric actuation presents distinct advantages, including its electrical sensitivity, compliance, and integration potential. However, IPMC actuators do face certain limitations, such as low power density and relatively modest stress levels. Additionally, issues related to restricted motion and force output, as well as challenges in modeling, control, and overall robustness, call for further investigation and refinement in the realm of IPMC actuators.

*SMM-based actuation:* SMMs have gained marked attention as a promising approach for soft actuation in the realm of soft robotics. These materials possess a unique property, the ability to revert to their initial configuration when exposed to certain stimuli, such as temperature or light. This exceptional feature enables SMMs to exhibit remarkable shape-changing capabilities, making them a prime choice for soft actuation across a wide range of robotic applications. SMM-based actuators mainly include shape memory alloy (SMA) actuators and shape memory polymer (SMP) actuators, as discussed below.

SMA threads are commonly employed in the design of compliant actuators. The application of an external electric field induces a current within the SMA circuit. When the Joule heat generated by this current surpasses the critical temperature of the SMA thread, it triggers a contraction force, resulting in the bending deformation of the elastomer [65]. Due to the shape memory behavior induced by the crystalline phase transition from martensite to austenite at elevated temperatures under electrothermal conditions, SMAs can effortlessly restore their original form. This crystalline phase transition-induced shape memory behavior allows the conversion of heat into mechanical motion, thereby facilitating the lightweight and streamlined realization of compliant actuators. Nickel-titanium is one of the most commonly used alloys for SMA actuation. For instance, Laschi et al. studied a soft arm inspired by the biomechanics of an octopus arm. This soft arm is actuated by a cable with an SMA spring. By energizing the spring at different parts, the tentacle can bend at multiple nodes, extending and contracting for the manipulation of objects [66]. Due to the inherent advantages of lightweight and convenience in SMA, Huang et al. have fashioned a cordless soft robot propelled by an SMA actuator powered by a compact battery. This innovative design enables the robot to emulate biological rolling and swift ascent on inclined surfaces (Fig. 2B) [67]. SMA actuation boasts high energy density and high stress, but it tends to produce smaller displacement deformation and operates at lower frequencies compared to other actuation methods.

SMP is a class of smart materials, many of which are thermally responsive and can be programmed to remember temporary shapes, returning to their permanent shapes when exposed to a specific stimulus, such as heat or light. SMP offers advantages such as high elastic deformation, low density, low cost, and ease of manufacture. For example, Ge et al. integrated thermally driven SMP microgrippers, designed through stereolithography. These microgrippers can be activated by adjusting the printed shape to achieve various states of closure and opening, responding to heating [68]. However, SMP actuation presents challenges, including low mechanical strength, low recovery stress, and short cycle life. To address these limitations, reinforced fillers can be used to improve mechanical properties and increase shape recovery stresses.

#### External stimulus actuation

External stimulus-based actuation strategies encompass magnetic response actuation strategies and electrical response actuation methods. These strategies exploit environmental stimuli such as light, heat, and magnetism to drive soft robotics. This section presents a unified overview of external stimuli-based actuation strategies, classifying them based on different external conditions.

*Moisture- and light-driven actuation:* Moisture-driven actuation relies on the absorption or desorption of water by a material, which, in turn, leads to a change in its volume or shape. Some soft materials, such as hydrogels or certain polymers, expand or contract when exposed to water, resulting in a change in volume or shape. Light-driven actuation utilizes the interaction of soft materials with light, usually achieved through light-responsive materials. When these materials are exposed to specific wavelengths of light, they can undergo reversible changes in structure, shape, or properties. CNTs exhibit a unique tubular structure with unique hybridized backbones, endowing them with extraordinary properties, particularly in the realm of photo response [69]. Zhou et al. introduced a moisture- and light-driven actuator based on CNT-coated paper and polypropylene composites. They created a smart gripper with an expandable initial opening width using the bi-directional bending motion of the actuator. This allows the gripper to increase its opening width up to 4 times its initial size, making it suitable for gripping larger objects [70]. Graphene, characterized by an ultra-broadband optical response spectrum and extremely strong nonlinear optical properties, has become a focal point in the development of optically driven soft actuators [71]. A soft actuator crafted from graphene, as depicted in Fig. 2C, harnesses structural absorption of radiant energy to induce its motion. Under the influence of light, electricity, organic vapors, and humidity, this actuator achieves a spectrum of programmable movements [72]. This actuation method not only has important environmental advantages, but also enables the designed robot to realize remote control with a certain degree of accuracy. However, this approach presents difficulties in performing kinetic modeling and certain challenges in designing structures based on photosensitive materials. Moreover, hydrogels or other photosensitive materials are prone to degradation over time, potentially impacting the lifetime of the robot.

*Thermal-driven actuation*: Thermal-based actuators play an important role in flexible actuators, and they are broadly categorized into photothermal actuators and electrothermal actuators based on the mechanism of the external thermal stimulus. Photothermal actuators primarily leverage the thermal expansion effect resulting from the localized temperature increase induced by light path irradiation. On the other hand, electrothermal actuators rely mainly on the Joule heat effect in combination with the special phase transition characteristics of the material to achieve deformation. Typical material choices include SMPs, liquid crystal polymers [73], and CNTs based on electrothermal response [74]. Yang et al. developed a bio-optical hose driver that utilizes polymerizable bio-optical hose actuators. This actuator takes advantage of the sensitive and photosensitive properties of the selected material [75]. When combined with liquid crystal elastomer (LCE), the hose actuator will bend and deform due to the material’s excellent photothermal conversion efficiency and the molecular arrangement changes caused by local temperature rise caused by near-infrared light. This innovative actuator demonstrates excellent adaptability to light sources and possesses the capability for omnidirectional light-tracking, promising applications in the field of soft robotics. Kobayashi et al. designed a gripper that responds to thermal cues. This soft gripper is not only biocompatible but also biodegradable, making it suitable for thermally driven drug patch applications. They also create a range of thermally responsive self-folding structures in response to 3 different temperatures, offering numerous prospects for future research into soft robotics [76]. Photothermal actuators enable remote and precise control, but their effectiveness may be limited by the depth of penetration. Electrothermal actuators are cost-effective and versatile, but heat dissipation and their limited spatial resolution are issues that need to be addressed.

*Magnetic-driven actuation:* Magnetic-driven actuation in soft robotics often involves the incorporation of magnetically responsive particles [77], including iron oxide, triton tetroxide, ferrite powder, and NdFeB [78]. For example, Ze et al. designed a soft robotic origami crawler that consists of a 4-unit special component with magnetic plates on each component, as shown in Fig. 2C. These plates are made of silicone and embedded with hard magnetic particles, and their susceptibility can be fine-tuned by controlling the volume fraction of the magnetic particles. A uniform magnetic field creates torque, causing the origami track to drive. This principle is used to facilitate the rotation and expansion of each special unit, which, in turn, enables the drive of the origami track [79]. One of the major problems with magnetic soft robotics is the lack of functionality, as well as the complexity of material design and fabrication methods to create deformable robots containing multiple curved structures. The researchers have constructed a modular soft magnetic architecture in which individual units contain rutile iron boron particles with a defined magnetization profile. These units are embedded in an adhesive network on both sides of a double-sided adhesive, allowing the mass production of magnetic soft robots with exceptional robustness, capable of withstanding loads thousands of times their weight [80].

In general, external stimuli offer the advantage of minimal physical contact, making them suitable for a wide range of micro- and small-scale robotic systems. However, they require careful consideration of the external environment and the choice of soft materials. Magnetic and optical actuation-based soft actuation strategies have been widely used in micro-robots, yet there is ample room for further exploration and advancement in this field.

#### Passive deformation

Compared to traditional motor actuation methods for rigid robots, the actuation techniques in soft robotics are notably more intricate. While the compliant structure itself is not a direct driving mechanism, it is designed to undergo deformation and propulsion through the influence of a passive mechanism. This dynamic occurs when external motors or cables, transmitting tension through tendons, prompt the deformation of the actuator, thereby propelling the soft robotic system.

*Cable-tendon actuation:* Cable actuation is a widely used external force-based actuation strategy in traditional flexible robots. The core principle involves passing a cable through a fixed point on the mechanical body and pulling a cable at its root to generate a certain bending moment at the fixed point, thus causing the body to move. Cable actuation stands as a superb method for achieving extended-distance transmission, particularly suited for the design of elongated soft robotic systems. For example, Zhou et al. [70] combined motors and cables to design a soft robot capable of planning and predicting motion trajectories, providing a more convenient actuation method compared to alternative strategies. This fusion of the motor and cable provides the soft robot with trajectory planning and prediction, streamlining the propulsion process. In the realm of intelligent equipment, cable-driven soft robots can generate substantial force, making them an excellent choice for medical rehabilitation devices. Delph et al. [81] have devised a cable-driven soft robotic glove (Fig. 2D) designed to assist stroke patients in practicing hand grasping exercises. In addition, such tendon-driven soft robots can also be used for the rehabilitation of patients with spinal cord injuries [82], which has high application value.

*Motor-tendon actuation:* Another uncomplicated driving method involves the collaborative action of a driving motor and tendons to achieve passive deformation. Elaborate deformations and motions can be attained by employing multiple motors, each connected to different regions of the soft structure through tendons. The motor-tendon driving mechanism bears resemblance to the biological interplay between muscles and tendons, making it particularly well-suited for intelligent wearable devices [25]. However, servo motors, while effective, have a marked drawback in that they are rigid and bulky components, limiting the softness and flexibility of the overall system. In the domain of soft robots, researchers have developed robots that utilize servo motors to achieve various actuation modes, such as flying and jumping. For example, Chang et al. modeled how birds manipulate their feathered wings during flight. With the help of servo motors, they developed a bio-hybrid wing with 40 un-actuated real pigeon feathers. This wing was strong, soft, and ultra-light compared to previously reported carbon-based and glass fiber-based robotic feather wings [83]. Furthermore, Fig. 2D illustrates a flexible motor-tendon gripper [84], employing cables as an intermediary drive to enable the gripper to capture objects of varying shapes and weights through different grasping techniques.

Although passive deformation strategies offer high efficiency in force transmission, they also bring about increased complexity and bulkiness within the actuation system. This complexity poses challenges when attempting to downsize soft robots. A potential solution to this issue lies in the pursuit of miniaturizing external driving components. Simultaneously, efforts to integrate diverse external driving elements directly into the robot’s main body can prove effective in streamlining the system and overcoming the challenges associated with complexity and size.

In summary, these actuation strategies have their strengths and weaknesses, making them valuable assets for specific robotic applications. For detailed information on the advantages and drawbacks of 5 different actuation strategies, please refer to Table 1. Additional details on multimodal actuation will be provided later.

**Table 1.** Comparison of actuation strategies

Actuation strategy		Mode of actuation	Actuation signal	Dynamic profile	Strain	Motion profile	Classic features	Ref.
**Pressure**	**Pneumatic**	Contraction and expansion	0–20 kPa; 0.066–1 Hz	7.5 N blocking force and 9.5 N pushing force	25%–30%	10 mm/circle	Highly versatile; high force transmission	[48]
	*Advantages:*							
	• High power density							
	• High variability							
	*Drawbacks:*	Contraction and expansion	0–4 bars	30–100 N axis force	10%–25%	100 mm under 0.4 bar	Soft recovery hand; high effective force	[161]
	• Massive size							
	• High sealing requirements							

		Bending and gripping	3-3 solenoid valves from 0–4 bars	17 N under 4 bars	50%	133° joint angle	Wearable hand recovery device; integrated actuation and sensing	[162]
	**Hydraulic**	Swimming, diving and turning	1.3 Ah battery under 72 MHz signal	Overcomes 1.65 kg of underwater resistance	Wide range deformation	100 mm/s	Prolonged underwater exercise; multimodal movements	[88]
	*Advantages:*							
	• High corresponding frequency							
	• High actuation force							
	*Drawbacks:*	Stretching	1.5 MPa or 2.5 MPa initial pressure	Up to 7 MPa	26%	230°/s	High stability and compliance	[163]
	• High-quality impact							
	• Hard to model and control							

**Electric**	**Dielectric elastomers (DEs)**	Crawling	1.2–1.6 kV; 1–150 Hz	22 mN block force under 1.6 kV	7.5%	56 mm/s	Sub-centimeter class pipes; multimodal movements	[90]
	*Advantages:*							
	• High actuation speed							
	• High strain							
	• High scalable	Swimming	7–9 kV, 2 Hz	8% axial strain under 110 actuation pressure	7.0%	32.9 mm/s	Movement in deep sea; long life span	[18]
	*Drawbacks:*							
	• High voltage required							
	• Poor flexibility and robustness							
		Bending	5 kV, 2–10 Hz	3.6 mN block force under 5 KV	More than 10%	47 mm under 3,500 V	Enables fast, large dynamic displacements	[164]
	**IPMC**	Bending	0–5.25 V	0.31 mN maximum load	None	12 mm deflection under 1 V	Excellent proton conductivity; fast actuation capability	[165]
	*Advantages:*							
	• Low operating voltage							
	• High working frequency							
	*Drawbacks:*	Bending and crawling	0–5 V; 2,000 Hz	35 μN under 5 V	10%	5 mm under 5 V	IPMC’s new manufacturing process	[166]
	• Low strain							
	• Low power density							

	**SMM**	Crawling and swimming	30 V heating voltage	Lifting a 0.91-kg loads	100%	140 mm/s	High response time; excellent adaptability	[167]
	*Advantages:*							
	• High energy density							
	• High stress							
	*Drawbacks:*	Straight turning and moving	0.65 A actuation current under 0.56 Hz	Maximum pulling force 0.98 N	21.5%	18.8 mm/s	Amphibious working ability; multimodal drives	[65]
	• Small range of motion							
	• Low actuation frequency							

**External stimulus**	**Magnetic**	Crawling	20–40 mT,1Hz	Small torques of 0.18 N.mm	30%	3 mm/s	Quick turn small-scale pipe movement	[79]
	*Advantages:*							
	• No external contact required							
	• Enables miniaturization of drive units							
	*Drawbacks:*	Crawling, locomotion	100 mT	Flexible contact, small block force	55%	4 mm under 1,200 circles	Multimodal robot motion	[80]
	• Sedimentation of magnetic particles							


	**Glossy**	Bending	808 nm near-infrared light irradiation	17 MPa stress under 0.2 wt%	120%	0.08 rad/s	High optical tracking accuracy	[75]
	*Advantages:*							
	• Low energy consumption							
	• Contactless work s							
	*Drawbacks:*	Bending and gripping	0.304 W/cm^3^ light illumination	Grasping a 2.8 g object	140%	13 mm under 20 s	Multi-actuation options; excellent mechanical properties	[95]
	• Low movement speed							
	• Strict material restriction							

**Passive deformation**	**Cable-tendon**	Bending and grasping	Direct current (DC) motor with 10 N loads	3 N maximum grasping force	90° angle rotation	70 mm/min	Gripping of fragile or irregularly shaped objects	[168]
	*Advantages:*							
	• Excellent force transmission							
	*Drawbacks:*							
	• Complex and large actuation system, difficult to miniaturize	Crawling	DC motor actuation	5 N blocking force	None	200 pixel movements	Excellent force transmission	[169]
									**Motor-tendon**	Bending	DC motor actuation with crank	Cable pulling force 1 N	*E* = 110 kPa	4.5–10 mm range	Suitable for exercise in a variety of environments	[170]
									*Advantages:*							
									• Simple and straightforward							
	*Drawbacks:*															
	• Rigid components limit system flexibility	Self-directed flight	Two dual-port DC motor with MCU	Maximum wing force 13 N/m^2^	None	10 Hz flapping speed	No-traction autonomous flight	[171]

### Multimodal actuation strategy

In the previous section, we introduce 4 typical actuation strategies for soft robots. While these individual strategies have shown significant results in specific contexts, they often come with limitations related to specific forms of motion and task requirements. To further enhance the flexibility and versatility of soft robots, researchers are now delving into multimodal actuation strategies [85]. Multimodal propulsion can be achieved through 2 distinct strategies. The first strategy involves employing a singular driving strategy, wherein a variety of propulsion modes can be generated through structural design or the combination of multiple actuators. This approach is commonly used in externally stimulated soft robots, as the stimulation source and threshold can be adjusted according to the target mode. Pressure-driven and electrically driven approaches also serve as commendable choices for achieving multimodal propulsion, relying primarily on the combination of multiple actuators in various regions of the elastomer, coupled with specialized structural designs to realize diverse driving modes. The second strategy involves the generation of multiple propulsion modes under the influence of various driving strategies. These strategies enable soft robots to exhibit multiple forms of motion within a single drive mode by combining different drive modes [86]. The amalgamation of typical diverse driving strategies is primarily grounded in the passive deformation of tendon-driven mechanisms, upon which pressure-driven, electrically driven, and externally stimulated drives are integrated to achieve multimodal motion. The key to this approach lies in effectively coordinating and regulating interactions between different actuation techniques to achieve a wide range of robotic movements.

**Fig. 3.**
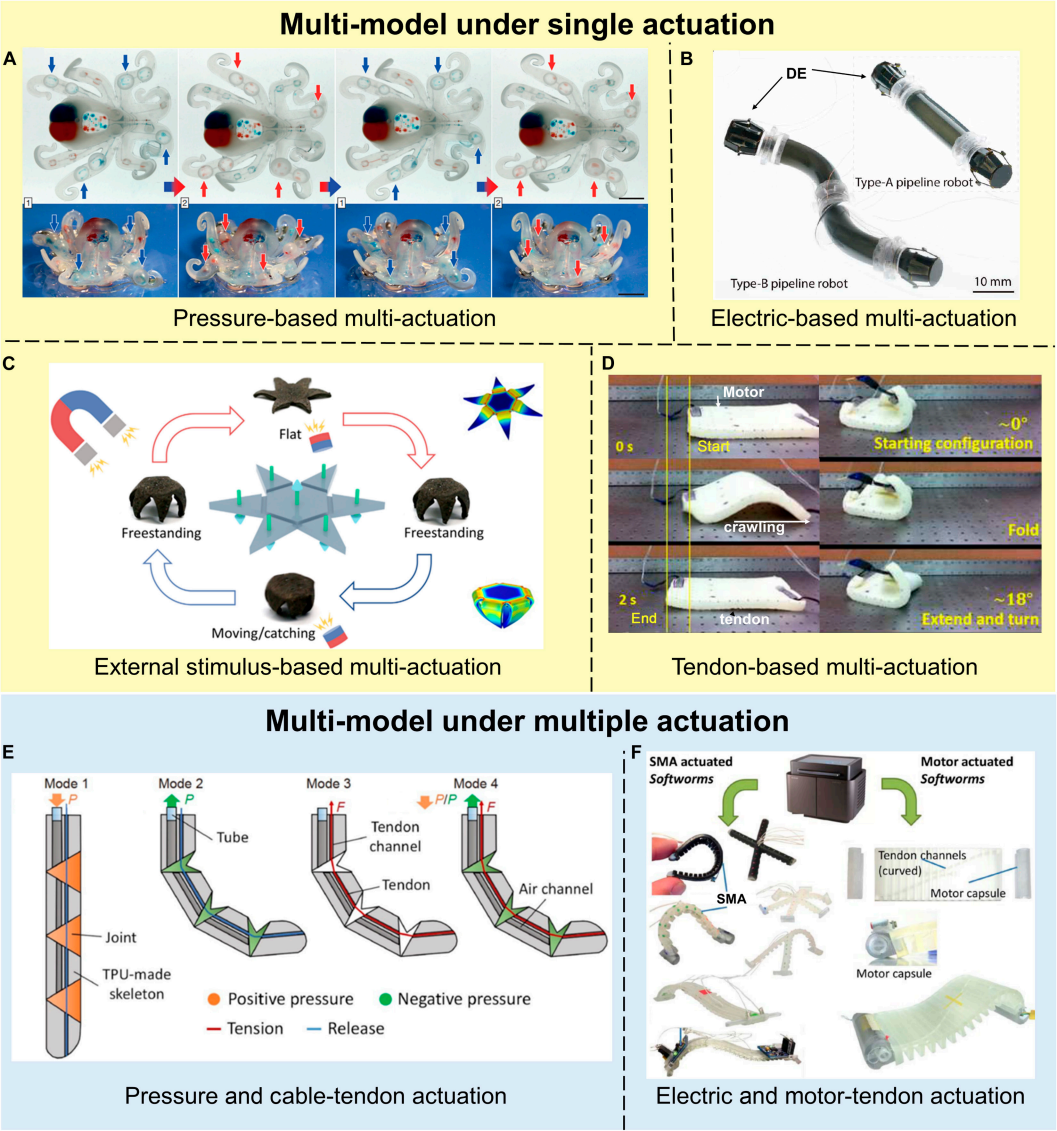
Multi-modal actuation strategies. (A) Pressure-based multimodal actuation. Reproduced with permission from [87]. (B) Electric-based multimodal actuation. Reproduced with permission from [90]. (C) External stimulus-based multimodal actuation. Reproduced with permission from [94]. (D) Tendon-based multimodal actuation. Reproduced with permission from [96]. (E) Multi-model under multiple actuation: Pressure and Cable-tendon coupled actuation. Reproduced with permission from [98]. (F) Multi-model under multiple actuation: Electric and motor-tendon coupled actuation. Reproduced with permission from [99].

Common forms of motion for soft robots include bending, but by tuning the distribution and control of the actuators, a range of other motions can be achieved, such as crawling, looping, rowing, tumbling, and swimming. Figure 3A shows an all-soft octopus’ robot, which, through the coupling of monopropellant fuel with microfluidic logic, enabled it to drive, control, and realize the autonomous operation of these pneumatic drive systems [87]. The soft controller’s oscillator causes the octopus robot to alternate between blue and red actuation states. In addition, Katzschmann et al. presented an autonomous soft-bodied robotic fish that was hydraulically driven and able to swim continuously in 3 dimensions. The combination of a gear pump, a hydraulic cylinder, and a soft-body tail enabled the soft-body fish to move forward and dive under water [88]. In this work, the multimodal movement is achieved through a combination of rigidity and flexibility. In addition, Bartlett et al. [89] designed a combustion-driven robot that is powered by butane and oxygen combustion. Moreover, the robot consists of a main explosive actuator surrounded by 3 pneumatic legs. This jump design also offers the possibility of working in some complex environments.

The emphasis on multimodal propulsion relying on a singular electric drive primarily revolves around 2 strategies: DEs and SMAs. These 2 actuators are favored due to their high energy density, making them particularly suitable for applications requiring versatile propulsion capabilities. Tang et al. designed a micro-robot for navigation in sub-centimeter pipeline environments (Fig. 3B). This micro-robot harnessed the properties of 2 DEs of different Young’s modulus. A rigid DE was strategically positioned at both the head and tail of the robot, functioning as anchoring points. In contrast, a softer DE was located in the middle portion of the robot, serving as the telescopic segment. The robot movement was achieved through a clever mechanism of alternating between anchoring and elongation of these DEs [90]. Benefiting from the SMA’s rapid response, high power-to-weight ratio, and compatibility with small and lightweight electronic devices, Patel et al. [91] strategically embedded SMA coils within the actuator. This integration enables the robot to rapidly transition between crawling and rolling gaits, adapting effortlessly to various terrain structures.

Magnetic propulsion stands out as a highly effective method for achieving multimodal propulsion, given the high penetrability of magnetic fields, enabling the actuator to realize unrestricted motion in unstructured environments [92]. Simultaneously, these actuators, in comparison to the preceding 2 types, are more adept at executing intricate movements, albeit with stringent requirements for the magnetic field. For instance, Li et al. designed a self-vectoring electromagnetic soft robot. Modularization and recombination of magnetron-based micro-regions allowed for diverse motions, including expanding rotation, left-right rocking, left-right flipping, and continuous rolling, achieved by applying different current signals [93]. This work demonstrates the multimodal motion of a soft robot through a combination of actuation methods and modularity. Sun et al. reported a magnetic micro-robot, which consists of multiple magnetic bundles with joints that can transform bending deformation into folding deformation to localize deformation in the joint region (Fig. 3C). It is capable of multimodal motions such as moving, flipping, grasping, carrying and releasing, and programmable shape transformations [94]. Similar to magnetically responsive actuators, photonic responsive actuators also allow for remote operation. However, their response time is comparatively sluggish, and their efficiency is lower. Li et al. [95] engineered a flexible gripper using nanocarbon-based polymers endowed with outstanding driving and optoelectronic capabilities. Through external light irradiation at 0.3 W/cm^2^, they achieved photo displacement-induced bending in composite actuators with varying thicknesses of CNTs.

Although the passive deformation strategy that combines motors, cables, and tendons imposes limitations on the miniaturization and mobility of devices, it possesses efficient force transmission characteristics. Through the combination of various actuators, it can still achieve multimodal propulsion for soft robots. For example, Kastor designed a motor-tendon-driven soft foam robot, integrating 2 motor-tendon actuators diagonally on the soft foam. This integration allows the robot’s body to compress or fold, enabling movements such as forward progression, turning, and flipping, as depicted in Fig. 3D [96]. Moreover, tendon-driven mechanisms are frequently employed in flexible wearable devices. Building upon tendon-driven multimodal propulsion, a dynamic model is constructed using finite element analysis. Leveraging reinforcement learning, the glove’s pose is optimized, endowing the glove with more dexterous grasping and convenient in-hand manipulation [97].

Several instances of multimodal actuation achieved through the coupling of various actuators are evident. Take, for example, the previously mentioned flexible gripper [95], showcasing the capability of photonic actuation while also accomplishing gripping tasks at low voltage. This gripper exhibits a natural open state, and upon applying a 15-V voltage, the free end bends inward, effectively securing the object. The multimodal motion of the soft robot is achieved by combining electrically responsive driving strategies with external photonic responsive driving strategies. As illustrated in Fig. 3E, Zhu et al. [98] introduced a pneumatic and tendon-coupled soft actuator with various driving modes. This design allows the robot to exhibit low output force and rapid response under pneumatic actuation, as well as high output force and flexible response speed regulation under tendon actuation. The structure is compact, and the force and speed range are extensive. The multimodalities constructed from 2 driving modes with different force and speed characteristics can cover a variety of application scenarios. This serves as a typical example of achieving multimodal actuation through the combination of various driving strategies. Additionally, electrically driven strategies can be combined with tendon-driven strategies to achieve multimodal actuation. Figure 3F demonstrates an integrated design of SMA actuation and motor-tendon actuation [99], realizing the crawling and rolling of a biomimetic soft worm.

Soft actuators play a fundamental role in achieving diverse movement objectives, including bending, stretching, and twisting [100]. The key to enhancing the intelligence of soft robots lies in achieving multimodal actuation. While each actuation strategy has its strengths, it also comes with inherent limitations. To achieve multimodal actuation, the focus often shifts toward hierarchical actuation within a single driving mode. However, this introduces complexities into the system and presents new challenges in terms of modeling, simulation, and control of soft robot motion. Pressure-driven actuation is one of the most well-established soft actuation strategies. However, due to the extensive involvement of external connection components, it can be challenging to attain modular multimodal motion. Conversely, electric actuation strategies and external stimulus-driven strategies benefit from their non-contact nature, which allows them to achieve multimodal actuation through coordinated cooperation among modules. Overall, achieving multimodal actuation is a crucial step toward realizing intelligent soft robots.

## Strategies for Soft Sensing

**Fig. 4.**
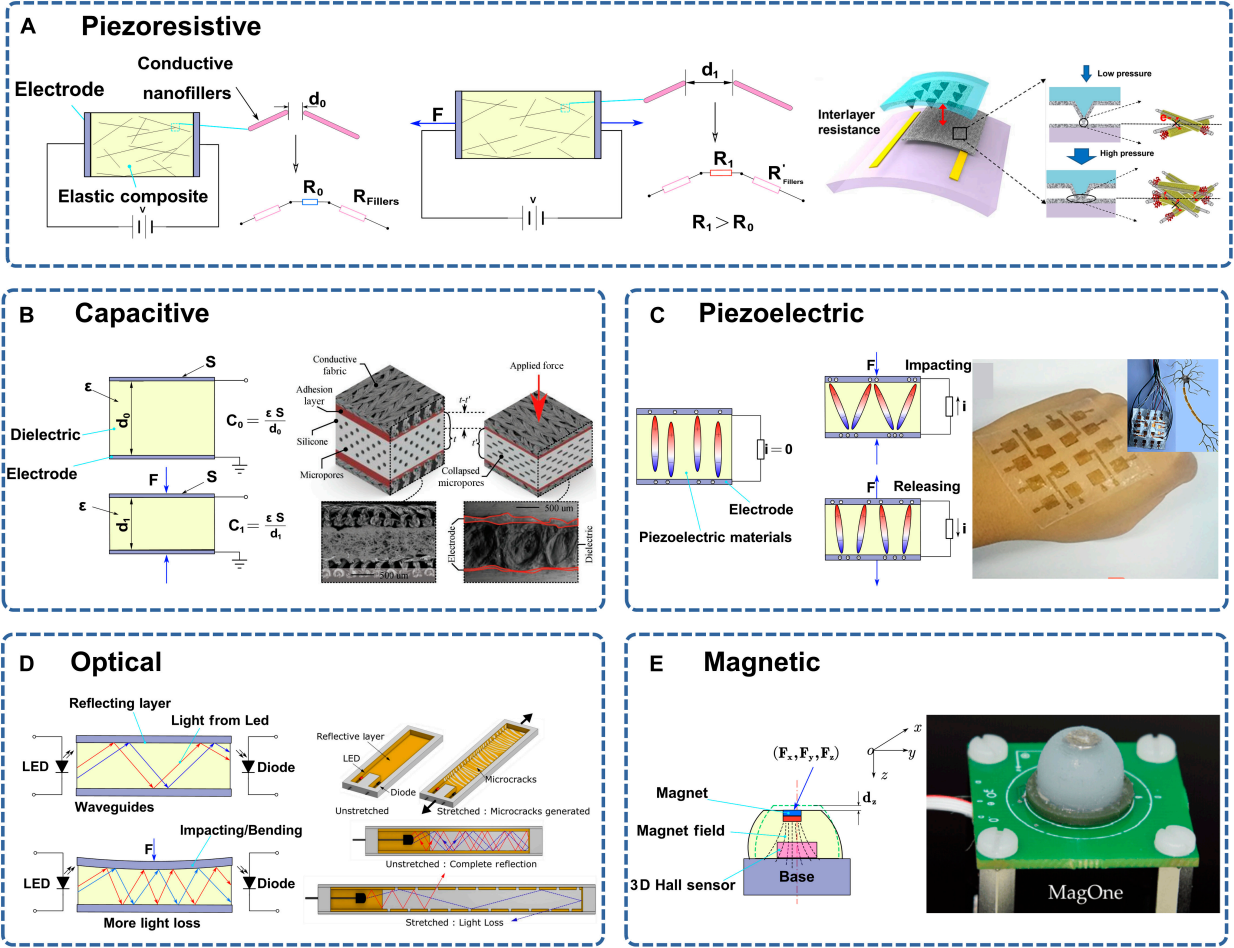
Various sensing mechanisms and representative designs for soft robotics. (A) Working mechanism of the piezoresistive sensor and a design with biomaterial-incorporated conductive interfacial layers. Reproduced with permission from [103]. (B) Working mechanism of the capacitive sensors and a representative design. Reproduced with permission from [160]. (C) Working mechanism of the piezoelectric sensor and a single-electrode e-skin. Reproduced with permission from [107]. (D) Optical sensing mechanism and a representative design. Reproduced with permission from [101]. (E) The working principle of the magnetic sensor and a tactile magnetic sensor. Reproduced with permission from [111].

**Fig. 5.**
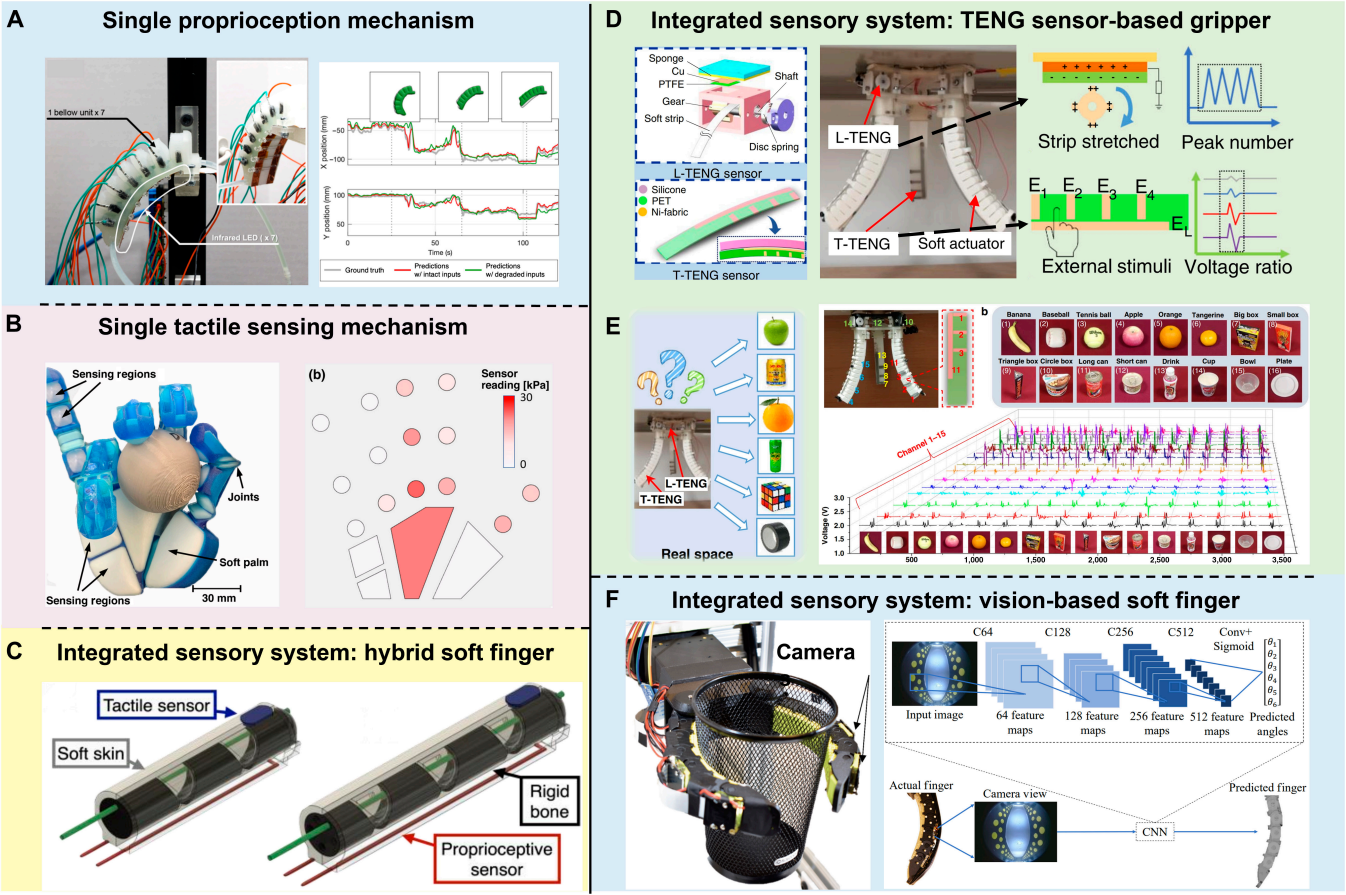
Single proprioception and tactile sensing mechanisms and integrated sensory systems. (A) A recurrent neural network (RNN)-based method for proprioception. Reproduced with permission from [116]. (B) A 3D-printed robotic hand for tactile sensing. Reproduced with permission from [119]. (C) Schematic of a highly integrated soft finger. Reproduced with permission from [120]. (D) The basic structures and data processing strategies of the length TENG (L-TENG) sensor and the tactile TENG (T-TENG) sensor. (E) The TENG-equipped soft gripper demonstrates distinctive voltage responses to various objects. Panels D and E are reproduced with permission from [113]. (F) A vision-based soft finger. Reproduced with permission from [117].

A defining feature of soft robotics is its capacity to achieve intricate movements through simple open-loop control. However, open-loop control alone cannot achieve high-precision operations and error correction. Hence, the integration of sensors to establish closed-loop control systems is essential for the advancement of soft robotics [101]. In this section, we will explore various sensing mechanisms, highlighting several representative works based on different sensing principles (Table 2 and Fig. 4). Furthermore, for a soft robot to autonomously perform tasks, it must possess the ability to perceive its shape (proprioceptive sensing) and detect stimuli from the external environment (tactile sensing). We will further introduce the concepts of proprioceptive and tactile sensing and present integrated sensors that combine both proprioception and external perception (Fig. 5).

**Table 2.** Overview of typical sensing mechanisms for soft robotics

Principle	Materials (electrodes)	Sensing functionality	Responsive speed	Sensitivity	Limitations
**Piezoresistive**	Nanocomposite, PDMS particles	Curvature, strain, elongation, contact force	Low	Low	Need to reduce hysteresis
**Capacitive**	Nanocomposite, hydrogels, textile	Curvature, bending angle, proximity, contact force	Fast	High	Need to minimize sensitivity to environmental contaminants
**Magnetic**	Permanent magnets	Curvature, elongation	Middle	High	Need to mitigate vulnerability to external interferences
**Piezoelectric**	PVDF, BaTiO_3_, PZT, ZnO	Curvature, elongation	Fast	Moderate	Need to enhance environmental adaptability
**Optical**	Optical fiber, waveguide, FBG	Curvature, elongation, bending angle	Fast	High	Need to reduce manufacturing costs
**Resistive**	Liquid metal, ionic liquid, nanocomposite, hydrogels, PDMS	Curvature, strain, bending angle, elongation, contact force	Low	Low	Need to improve sensitivity
**Inductive**	Coils	Elongation, bending angle,	Middle	Moderate	Need to simplify signal conditioning electronics
**Triboelectric**	PDMS, PTFE	Curvature, strain, elongation, contact force	Fast	High	Need precision manufacturing

### Sensing mechanisms based on different principles

Soft robotics encompasses a wide range of actuators and complex operational requirements, making it challenging to design a universal sensing system. Therefore, it is crucial to explore sensors based on different principles to meet specific needs. In this section, we will summarize various sensor types, compare their advantages and limitations, and provide an extensive overview of typical applications associated with each sensor type.

#### Piezoresistive and resistive sensors

Piezoresistive sensors are typically constructed from elastic composite materials infused with conductive nanofillers. When exposed to pressure, these sensors undergo changes in resistivity and geometric shape. As the working mechanism shown in Fig. 4A, these conductive nanofillers are interconnected, forming a network that carries electricity. Initially, with no pressure applied, the nanofillers are at a certain distance apart, providing a specific electrical resistance. When pressure is applied, the composite material stretches, causing the nanofillers to move further apart (the lower part of Fig. 4A). This increase in distance between the nanofillers disrupts the electrical pathway, increasing the overall resistance of the material. The alteration in resistance is a direct response to the applied force, allowing the sensor to detect and measure changes in pressure. The concept is visually depicted on the right side of Fig. 4A, illustrating how the network of nanofillers expands and subsequently influences the electrical properties of sensors. While these nanocomposite materials offer tunable mechanical and electrical properties, they often exhibit substantial hysteresis and nonlinearity. For example, Wang et al. [102] utilized MXene material to fabricate a piezoresistive sensor, involving the preparation of MXene material, the synthesis of MXene/polymer composites, and sensor fabrication. This sensor demonstrated high sensitivity, good stability, and repeatability, effectively functioning under bending and stretching conditions. However, it exhibited a narrow linear response range and important hysteresis, indicating the need for further improvement. In another example, Chang et al. [103] designed a piezoresistive sensor structure incorporating biomaterial-infused conductive interfacial layers, specifically using single-walled CNTs and M13 biological material (Fig. 4A). The fabrication involved mixing the single-walled CNTs and M13 biological material, printing them onto a polydimethylsiloxane (PDMS) substrate, and coating them with metal electrodes. This piezoresistive sensor demonstrated high sensitivity, rapid response, and good stability. However, the potential constraint on the broad applicability of these sensors arises from their heightened sensitivity, particularly within more constrained pressure thresholds.

Resistive sensors, similar to piezoelectric sensors, detect alterations in resistance resulting from variations in the geometric shape or resistive properties of conductive materials. A type of resistive sensor based on reduced graphene oxide and polyurethane sponge has been introduced, exhibiting notable characteristics such as high sensitivity and flexibility. This sensor’s design enables easy shaping into various forms to meet specific requirements [104]. In general, resistive sensors offer several advantages, including their simple structure, low cost, ease of fabrication and utilization, and the ability to measure diverse physical quantities such as deformation, strain, and curvature. However, it is essential to acknowledge their inherent limitations. Resistive sensors typically exhibit lower sensitivity and accuracy when compared to other types of sensors, and they are susceptible to the influence of environmental factors. Nonetheless, resistive touch sensors have gained popularity due to their affordability, rapid response, linear output, and high durability. It is worth noting that resistive sensors require a power source, rendering them unsuitable for low-power systems. In human signal detection, resistive sensors are commonly employed for measuring respiratory patterns, pulse signals, finger movements, and heart rate. In the field of soft robotics, resistive sensors facilitate the monitoring of deformations and movements, enabling adaptive control and intelligent perception. However, the lower sensitivity, limited accuracy, and susceptibility to environmental factors such as temperature and humidity should be considered when utilizing resistive sensors.

#### Capacitive sensors

Capacitive sensors are used to measure changes in capacitance resulting from variations in the geometric shape of elastic materials. They are employed for measuring physical quantities such as curvature, bending angle, proximity, and contact force. Capacitive sensors typically consist of 2 electrodes and a dielectric material, with changes in electrode spacing, dielectric constant, or charge storage capacity leading to corresponding changes in capacitance. When pressure is applied, the distance between the capacitor plates decreases, leading to a change in the distance between them (Fig. 4B). This change in distance results in a modification of the capacitance value. By monitoring the charge or voltage of the capacitor, real-time changes in the measured physical quantities can be tracked. Capacitive sensors offer benefits such as high sensitivity, rapid response, and good linearity, but they are susceptible to environmental pollutants. These sensors have widespread applications in fields such as soft robotics, e-skin, position, and shape detection [38].

For example, a stretchable capacitive sensor designed for tactile detection has been introduced [105]. This sensitization approach focuses on enabling proximity and tactile detection, which are fundamental components of a soft robotic finger. In another example, Atalay et al. introduced a wearable soft pressure sensor for electronics (Fig. 4B) [103]. The sensor features conductive fabric electrodes for reliable connections and a microstructured silicone dielectric layer to enhance baseline capacitance, reducing parasitic capacitance and improving the signal-to-noise ratio. The study explores the impact of different textiles and microstructural morphologies on sensor performance, emphasizing a conductive knit electrode and higher porosity for increased sensitivity. Integrated into a glove, the sensor measures grip pressure during object manipulation, offering advantages of ease of fabrication, flexibility, and secure connections.

#### Piezoelectric sensors

Piezoelectric sensors measure mechanical stress or pressure by utilizing the piezoelectric effect, which generates electric charges proportional to the magnitude of the applied stress or pressure. Figure 4C shows the working mechanism of piezoelectric sensors. When force is applied, the piezoelectric materials compress, inducing a charge. Conversely, when the force is released, the materials resume their original shape and the charge diminishes. These induced charges can be measured by the corresponding circuit. These sensors offer advantages such as high precision, high sensitivity, fast response, and wide-ranging applications. However, they are also susceptible to environmental factors such as temperature and humidity and require an external power supply. In the field of soft robotics, piezoelectric sensors find applications in posture perception, tactile feedback, and the measurement of mechanical stress and pressure.

Piezoelectric sensors also demonstrate marked potential for applications in self-powered physiological monitoring and human–machine interaction. A high-sensitivity and stretchable piezoelectric sensor has been proposed, utilizing finite element analysis and 3D printing techniques [106]. The strengths of their work are particularly evident in the straightforward fabrication process and the sensor’s remarkable sensitivity and stretchability. Nevertheless, further advancements could be made to enhance the energy conversion efficiency of the sensor. Another study explores the creation of e-skin that imitates human sensory functions using a piezoelectric nanogenerator made from electro-spun polyvinylidene fluoride (PVDF) nanofibers (Fig. 4C). This sensor can detect pressure and temperature variations with unique signals, enabling the simultaneous sensing of different stimuli. This advancement could lead to simpler, more effective artificial skins for use in robotics and prosthetics [107].

#### Optical sensors

Optical sensors are designed to detect changes in light resulting from strain or pressure applied to optical transmission media. These sensors measure changes in light properties such as intensity, frequency, or phase, to determine the extent of the applied strain or pressure. In Fig. 4D, sensors detect strain or pressure by monitoring alterations in light within the waveguides. Applied pressure or bending alters the light path, leading to light loss, and the sensors utilize this change to quantify the degree of strain. They offer distinct advantages, including high deformability and resistance to interference and contaminants. Optical sensors have found applications in diverse fields, including soft robotics, wearable pneumatic gloves, and prosthetic hands [108]. They play a crucial role in providing tactile perception in surgical manipulators and prosthetic fingers. A noteworthy feature of optical sensors is their ability to eliminate the need for electronic components and wiring in the sensing area. However, it is important to note their drawbacks, which include high manufacturing costs and a reliance on advanced signal conditioning devices.

A recent innovation in optical sensor fabrication involves the use of a highly reflective thin silver layer as an internal reflection layer (Fig. 4E) [101]. This method entails uniformly coating an elastic polymer surface with a thin silver layer. The resulting optical sensor exhibited exceptional stretchability and repeatability, because the deposition of the silver layer did not compromise the mechanical properties of the polymer during repeated loading-unloading cycles. In another study, an experimentally used fiber optic shape sensor (FOSS) was fabricated, which incorporated optical frequency domain reflectometry and utilized a specialty multicore fiber, integrating 4 optical cores within a single, monolithic glass fiber structure [109]. The FOSS system utilizes this multicore fiber to accurately monitor bending and twisting, providing detailed 3D shape assessments through sensors spaced at short intervals. It boasts high flexibility and a small diameter, enabling easy integration into various materials and maintaining performance under shape changes.

#### Magnetic sensors

Magnetic sensors utilize various transducer mechanisms, including coil geometry, mutual inductance, eddy-current effect, and magnetic reluctance, to measure changes in inductance [110]. They are employed to monitor the deformation, strain, displacement, pressure, or dimensions of soft actuators. The illustration in Fig. 4F demonstrates the response of an elastomeric material to applied external forces. The force applied leads to the displacement of an embedded magnet, and the changes in the magnetic field are subsequently detected by the Hall sensor. This sensor is capable of measuring the displacement of the magnet in 3 dimensions. These sensors offer advantages such as cost-effectiveness, high sensitivity, and easy integration. However, they are susceptible to fluctuations in external magnetic fields and can be affected by ferromagnetic objects [44]. Magnetic sensors find applications in diverse fields, including soft robotics, actuators, industrial manipulation, underwater exploration, and noninvasive medical procedures.

For example, a representative magnetic sensor was developed based on the principles of magnetic field induction by using magnetic-sensitive materials and highly elastic elastomers [111]. The fabrication process involved material mixing, injection molding, and magnetic field calibration. This sensor possessed the characteristics of durability, low cost, high precision, and high bandwidth, but with limitations such as nonlinearity and cross-interference. In addition, Alfadhel et al. introduced a magneto-resistive tactile sensor capable of operation in high-temperature environments, reaching up to 140 °C. This sensor employs bio-inspired structures known as cilia, which are fine structures fabricated from a magnetic nanocomposite material covering a spin-valve giant magnetoresistance sensor [112]. The magnetic nanocomposite material, composed of iron nanowires embedded in PDMS polymer, exhibits exceptional flexibility, biocompatibility, and high remanence. Moreover, it demonstrates robust adaptability to harsh environments.

#### Other sensors

In addition to the sensor types previously discussed, various other sensor technologies find common applications across a range of fields including triboelectric, ultrasonic, pneumatic, and inductive sensors. Triboelectric sensors rely on the frictional effect to generate electrical potential signals through physical contact, eliminating the need for an external power source. They offer advantages such as low cost, self-powering capability, wearability, lightweight, and comfort [113]. Ultrasonic sensors utilize the reflection of ultrasonic waves to detect the position of objects and the distance from the ultrasonic source. They are distinguished by high precision and non-contact measurement capabilities. Pneumatic sensors function by detecting the deformation and force of objects through changes in gas pressure. They are particularly suitable for deformation and tactile perception in soft robotics applications. Inductive sensors are employed to measure variations in inductance caused by factors such as coil geometry, mutual inductance, eddy current effects, and magnetic resistance [114]. They offer the advantages of low cost and high performance. Nevertheless, their drawback lies in the requirement for complex signal conditioning equipment.

### Hybrid sensing techniques for soft robotics

Self-perception and external perception play a pivotal role in the advancement and application of robots. Self-perception, also known as proprioceptive sensing, refers to a robot’s ability to perceive its state and position, encompassing joint angles, shape, deformations, and other relevant information. This capability empowers robots to execute tasks with enhanced precision, avoid collisions and damage, and adapt effectively to diverse environments and tasks [26]. Conversely, external perception, including tactile sensing, enables robots to perceive environmental information such as the position, shape, and texture of objects. This perceptual ability facilitates better interaction between robots and their surroundings, enabling them to undertake more complex tasks such as navigating different terrains, object recognition, and grasping. Hence, both self-perception and external perception are of paramount importance for the intelligence and practicality of robots. The previously described sensor technologies can be utilized to enable proprioceptive and tactile sensing capabilities in soft robotics [44].

#### Proprioception

Proprioception, or the sense of proprioceptive perception, refers to the ability of robots or organisms to control their behavior by perceiving their motion, position, and state [115]. In the context of soft robotics, proprioception plays a crucial role in achieving accurate task execution. It involves measuring the shape, posture, and deformation of the soft robot to enable closed-loop control. A proprioception method has been introduced, employing a hybrid sensor–actuator system within the fluidic medium of a soft robot (Fig. 5A**)**. Flexible electrodes detect changes in the robot’s shape, and a recurrent neural network (RNN) processes this data for accurate self-awareness of the robot’s position and movement [116]. Soft robots, with their infinite degrees of freedom and pliability, heavily rely on proprioceptive feedback to accurately perceive their body configuration. To achieve proprioception, various sensors are integrated into soft robots, including pressure sensors, strain sensors, gyroscopes, and accelerometers [44]. These sensors assist in perceiving the robot’s morphology, posture, and motion, enabling precise control and manipulation. Additionally, advanced techniques such as machine learning and deep learning are applied to enhance proprioceptive capabilities in soft robotics [117,118]. The role of proprioception encompasses precise control, adaptation to different environments and tasks, autonomous navigation, and obstacle avoidance. For example, in the biomimetic soft robotics field, proprioception principles draw inspiration from the neural systems of biological organisms, incorporating biomimetic learning and distributed neural control systems to achieve self-perception and adaptive control, thereby improving the motion controllability and environment adaptability of robots.

#### Tactile sensing

Tactile sensing plays a crucial role in soft robotics by enabling the perception of external stimuli. Soft robots are equipped with a variety of sensors, such as pressure sensors, capacitive sensors, and optical fiber sensors, which are embedded or coated on their surfaces to achieve tactile perception. These sensors are adept at detecting contact forces, deformations, and shapes when the robot interacts with its external environment. By utilizing tactile perception, soft robots can better perceive and understand their surroundings, ultimately enabling more precise control and operation. In Fig. 5B, a tactile sensing approach for soft robotics is developed, integrating pneumatic sensors into a 3D-printed robotic hand for environmental interaction. These air-filled sensors are placed within the fingers and palms to detect contact and pressure changes, enabling the hand to respond to physical touch using materials designed for sensitivity [119].

#### Integration of proprioception and tactile sensing

In the realm of soft robotics research, most studies have primarily focused on proprioception, with limited attention given to tactile sensing. However, it is essential to explore the integration of both proprioceptive and tactile sensing to gain a comprehensive understanding of the robot’s interactions with its environment. Following the introduction of the single proprioception mechanism **(**Fig. 5A) and tactile sensing mechanism (Fig. 5B), we have provided a comprehensive overview of prevalent sensing strategies that integrate both proprioception and tactile sensing. As shown in Fig. 5C to F, the integrated sensing strategy has been applied in soft robotics to develop soft fingers (Fig. 5C and F) and grippers (Fig. 5D and E). For instance, Georgopoulou et al. [120] have presented a highly integrated soft robotic finger capable of proprioception and tactile sensing by integrating various sensors, including muscle, pressure, and tactile sensors (Fig. 5C). In another example, the perception of texture and hardness was achieved by inferring tactile information through the installation of tension sensors on the tendons of the fingers [121].

Furthermore, researchers have introduced a soft finger utilizing the Gel Flex method, a vision-based proprioceptive and tactile sensor for soft robots. Inspired by the previous Gel Sight sensing technique, this approach involves creating an innovative exoskeleton-covered soft finger with embedded cameras. Deep learning techniques are then employed to achieve high-resolution proprioceptive sensing and extensive tactile sensing (Fig. 5F) [117]. These sensors enable the detection of finger position, deformation, and external force magnitude and direction, enabling self-perception and tactile sensing of the finger. This study further explores methods to address issues such as sensor signal drift and relaxation behavior by optimizing sensor materials, positions, and joint designs. Advancing the exploration of vision-based integration systems in soft robotics, another study compares shape reconstruction based on proprioceptive tactile sensing with one based on visual tracking [118]. This experiment involved recording the tactile responses and corresponding 3D shapes of the soft continuum finger using a spatially arranged capacitive tactile sensor array. The results demonstrate that proprioceptive awareness can be achieved in all 3 spatial axes, enabling body structure reconstruction and posture inference for the soft head, with an average accuracy of approximately 1 mm compared to visual tracking. Another study also explored the utilization of visual-based techniques for soft body self-perception, employing an embedded camera and convolutional neural networks to capture and represent deformation states, enabling accurate 3D shape reconstruction without external sensors [122].

A soft robotic gripper offers the advantages of more complex and integrated manipulation capabilities compared to individual soft robotic fingers. For example, 2 sensors, the length triboelectric nanogenerator (L-TENG) and the tactile TENG (T-TENG), enable proprioception and tactile sensing through triboelectric output signals generated from the contact and separation of electropositive and electronegative materials (Fig. 5D) [113]. The L-TENG sensor accurately measures the bending angle of soft actuators, while the T-TENG sensor effectively detects the sliding, contact position, and gripping mode of the soft gripper through patterned-electrode tactile sensing. This capability enables the gripper to accurately identify 16 different objects (Fig. 5E) [113]. In another example, Homberg et al. [123] presented a gripping mechanism employing a pneumatic system that incorporated composite sensors to achieve self-perception and tactile sensing. Each finger of the gripper was connected to a pneumatic piston via a tubing system integrated along the arm. Such gripping design allows for efficient and precise manipulation with the ability to perceive and respond to external stimuli through the composite sensors. Additionally, Zuo et al. developed a soft robotic gripper equipped with ionic hydrogel-based sensors designed for strain and tactile sensing, enabling object recognition through a learning-based approach. The hydrogel-based sensors exhibit exceptional conductivity, high stretchability, toughness, ambient stability, and a distinctive anti-freezing property [124].

Several other studies have employed diverse sensing strategies to achieve proprioception and tactile sensing. A soft robot arm with embedded tactile sensors at the joints has been designed to achieve self-perception. The tactile sensors were instrumental in acquiring self-perception and external environmental information, enabling pose estimation of the soft robot arm through machine learning algorithms [125]. Soter et al. [126] used an octopus-inspired arm as an example, where the proprioceptive representation is approximated by 4 bend sensors integrated into the soft body, presenting a novel method to implement bodily awareness into a real soft robot by the integration of its exteroceptive and proprioceptive sensors. Spielberg et al. [127] introduced a neural architecture that processed on-board sensor information to learn a salient and sparse selection of placements for optimal task performance. Their model and learning algorithm were evaluated on 6 soft robot morphologies for various supervised learning tasks, including tactile sensing and proprioception.

## Integrated Actuation and Sensing

Actuation and sensing are 2 crucial aspects of soft robots that importantly influence their locomotion, perception, and intelligence. To enhance the performance and functionality of soft robots, seamless integration of actuation and sensing has been dedicated to development, with the ultimate goal of achieving actuation-sensing integration. This section will focus on the significance and potential of actuation-sensing integration, including 2 main integration approaches: surface integration of sensors within the actuators and internal integration of sensors within the actuators. By closely integrating sensors with actuators, soft robots can achieve higher levels of perception and feedback, thereby enhancing their autonomy and adaptability.

### Surface integration

The development of e-skin comprising various flexible sensors such as strain, pressure, shear force, and temperature sensors, integrated with sophisticated circuits, has emerged to mimic the sensing functionalities of human skin [128]. These tactile stimuli are typically converted into electrical signals, such as changes in resistance or capacitance, which can be collected and processed by computers [32]. Tactile sensors are primarily classified into strain, pressure, and shear force sensors, depending on the targeted stimuli. In addition to tactile sensing, e-skin often incorporates temperature sensing, which provides early warnings to mitigate the risks of high- or low-temperature damage. Flexible temperature sensors have become an essential component of e-skin. Lastly, to approach the functionality of real skin, e-skin may incorporate humidity/solvent sensors to endow it with the ability to detect humidity or solvents.

With the continuous advancement of flexible electronics technology and e-skin, coupled with the introduction of more advanced manufacturing processes, the integration of high-performance actuation and sensitive sensing capabilities in soft robots has become feasible. For instance, Alessandro et al. proposed a novel optical flexible pressure sensor that consisted of 8 infrared emitters coupled with 8 photodetectors embedded in a flat PDMS waveguide with a diameter of 5.5 cm. This e-skin enables the detection of curved surfaces and mass in the gram range, providing a new solution for the realization of integrated actuation and sensing [129]. Similarly, Georgopoulou et al. used self-healing sensor fiber composites as e-skins on a 3D-printed soft actuator module to monitor the bending process. The change in relative resistance before and after injury allows the sensor to detect the actuator movement before and after healing [130]. Additionally, Sengupta et al. [131] reported a class of wearable, seeable, and sensitive piezoresistive sensors, which were fabricated by carbonizing electro-spun polyacrylonitrile (PAN) nanofibers and embedding them into PDMS elastic films. This approach enables both intrinsic proprioception and tactile sensing capabilities of the flexible sensor, which is further integrated into wearable devices for real-time monitoring of human movements, including gait analysis, wrist motion tracking, and respiratory monitoring, among other applications.

**Fig. 6.**
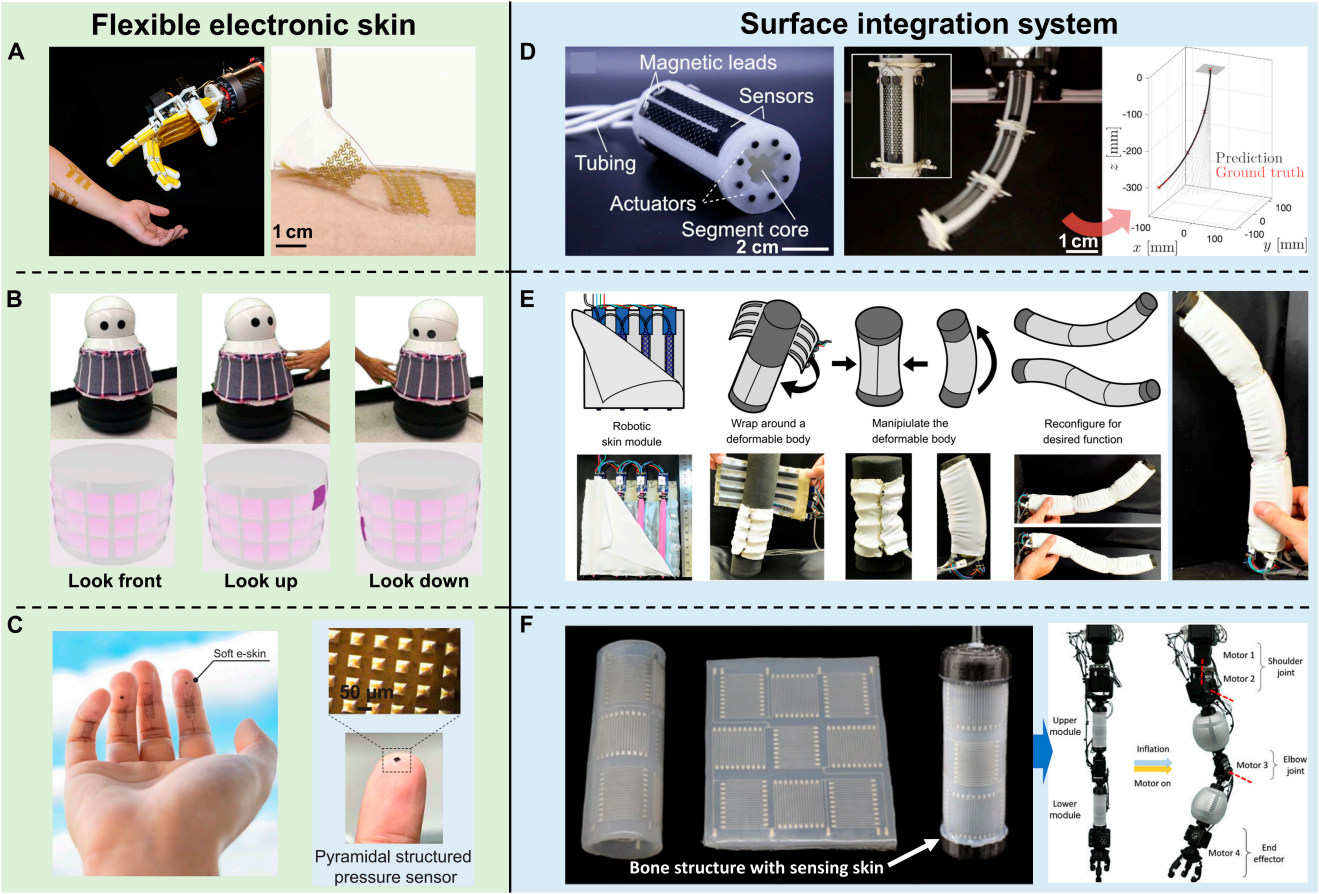
Surface integration of sensors in the actuation. (A) An all-printed mass-producible soft e-skin-based human–machine interface. Reproduced with permission from [132]. (B) A sweater-like tactile e-skin was applied on a Kuri robot for human–robot interaction. Reproduced with permission from [133]. (C) A low-voltage-driven artificial soft e-skin system. Reproduced with permission from [134]. (D) Piezoresistive sensors embedded in pneumatically actuated robot surfaces combined with predictive neural networks for proprioception. Reproduced with permission from [135]. (E) Omni Skins-based actuation-sensing integration strategy. Reproduced with permission from [136]. (F) Liquid metal-based microchannel e-skin for actuated sensing integrated robotic arms. Reproduced with permission from [137].

Several other studies have employed various methodologies to fabricate flexible e-skin. For example, a mass-producible soft e-skin with tactile pressure and chemical sensors, all fabricated through inkjet printing, was developed for robotic physicochemical sensing (Fig. 6A). The integration of AI-powered robotic control enhances the potential applications of this all-printed soft human–machine interface, extending to advanced robotic functionalities in automation, hazardous chemical detection, and human–robot interaction [132]. In another example, Si et al. developed a customizable machine-knitted tactile e-skin named RobotSweater, and applied it to a Kuri robot for human–robot interaction (Fig. 6B). Comprising multiple layers, including a mesh layer and conductive stripes forming a resistor matrix, this tactile skin offers robust contact detection, multi-contact localization, and pressure sensing. The fabricated sensor allows closed-loop control with tactile feedback, enabling human lead-through control of a robot arm and facilitating human–robot interaction with a mobile robot [133]. Additionally, in Fig. 6C, Wang et al. introduced a soft e-skin with a neuromorphic sensorimotor loop, featuring low-voltage, monolithically integrated sensor arrays for high-resolution tactile sensing and feedback control. The study highlights the design, fabrication, and sensorimotor capabilities of the e-skin, showcasing its capacity to detect and localize multiple contacts under various shapes [134].

As previously discussed in the “Strategies for Soft Actuation” section, various strategies for integrating actuation and sensing have been explored, which are the keys in achieving intelligent soft robots. One of the main approaches is through the integration of flexible e-skin with different actuation strategies. Turby et al. demonstrated the integration of a distributed soft proprioceptive sensor on a pneumatic-driven soft robotic arm, as shown in Fig. 6D. The integration of the pressure-based pneumatic actuation strategy and the flexible e-skin was achieved through plasma treatment. The deformation-induced changes in resistance were converted into a voltage, allowing for continuous real-time monitoring of the soft robotic arm’s shape [135]. While this approach focuses on proprioceptive sensing in a single actuation state, it enables the prediction of future deformations for convenient control. In Fig. 6E, Booth et al. presented a reconfigurable and drivable e-skin, which integrated both distributed actuation elements and sensors into a compliant substrate, wrapped around the soft body of the robot, enabling body movements. This approach is based on the integration of pneumatic actuation and capacitive strain sensors. Although it only achieves proprioceptive sensing, it simultaneously enables a variety of modalities in motion, such as rowing and caterpillar-like locomotion, based on the same e-skin. One notable feature of this work is the ability to impart multidimensional motion to a soft material [136]. Additionally, Kim et al. proposed a soft inflatable module with independent tactile sensing functionality, as shown in Fig. 6F. They incorporated a soft skin embedded with microchannels around the gas-driven module. By converting the pressure changes into voltage signals, proprioceptive sensing was achieved, and the module was integrated into a conventional rigid robotic arm for collision protection [137].

The actuation-sensing integration strategies for flexible e-skin primarily focus on pneumatic actuation strategies. However, they generally only achieve a single actuation mode, and successful integration of proprioceptive and tactile sensing in intelligent devices is relatively rare. Overall, there is still much room for further research in the field of actuation-sensing integration based on flexible e-skin. This mainly includes exploring the integration of other actuation strategies, achieving multi-modal actuation capabilities, and integrating proprioceptive and tactile sensing. Future efforts should be directed toward enhancing the stretchability of flexible e-skin while ensuring sensor sensitivity, thereby achieving more intricate integration of actuation and sensing.

### Internal integration

Beyond flexible e-skins, another typical strategy for achieving actuation-sensing integration is the internal integration of flexible sensors in the actuator (e.g., low elastic modulus elastomers combined with liquid-phase materials), allowing the flexible actuator to move more freely and flexibly. As a key component in sensing the external environment and its state, sensors provide the necessary feedback and information for the robot to achieve precise actuation control and intelligent interaction.

**Fig. 7.**
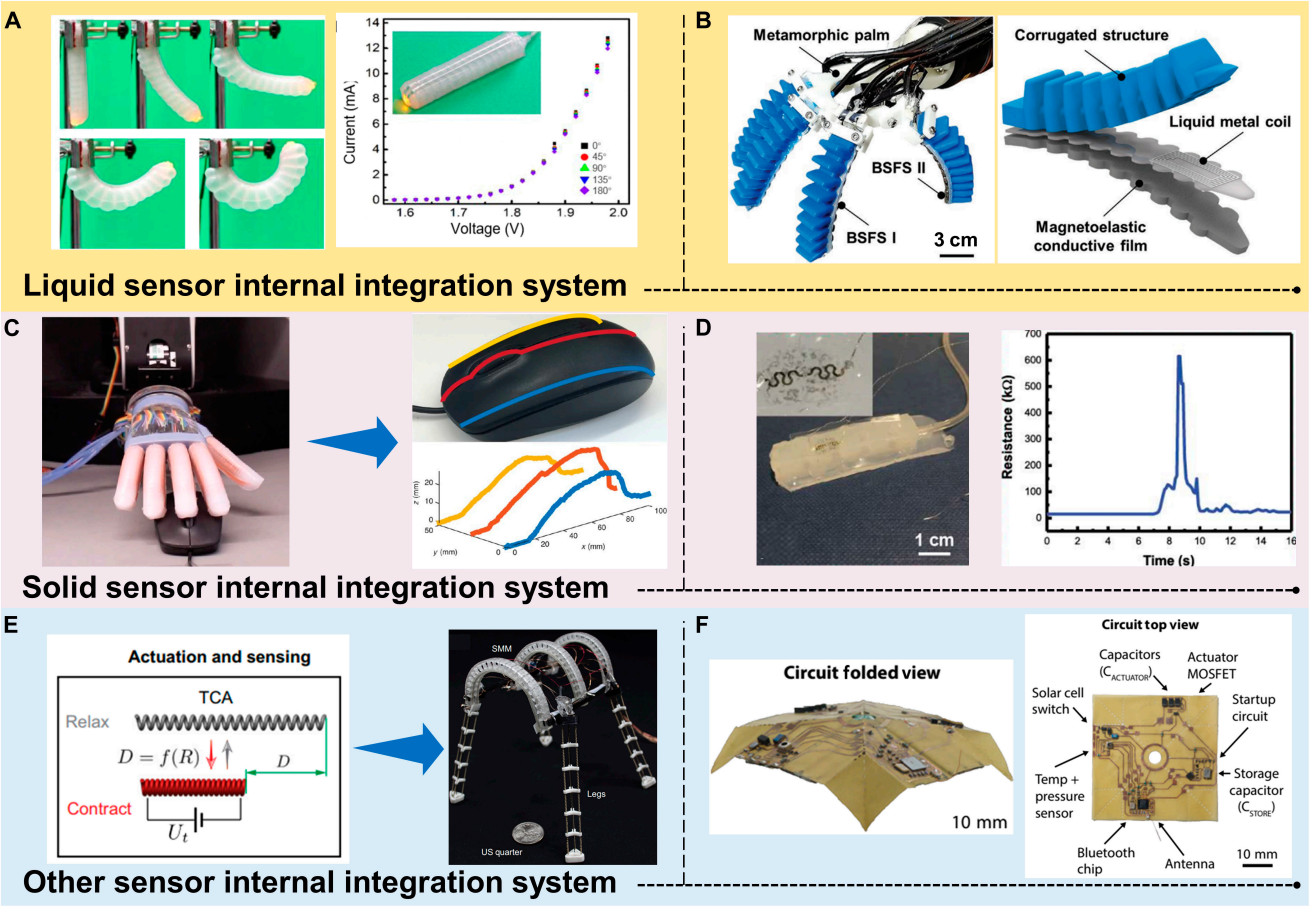
Internal integration of sensors in the actuation. (A) Nano-Fe Ga In mercury alloy for Smart Flexible Pneumatic Actuator. Reproduced with permission from [139]. (B) Structure of a flexible manipulator with bimodal self-powered flexible sensors (BSFSs) and a pneumatic soft finger with a BSFS as the base surface. Reproduced with permission from [140]. (C) Integration of stretchable optical waveguides with pneumatic fingers for tactile sensing. Reproduced with permission from [142]. (D) Flexible mechanical fingers after integration of stretchable strain sensors. Reproduced with permission from [143]. (E) Morphologically adaptive robots are realized with the bending angle of the robot body relative to time during deformation. Reproduced with permission from [144]. (F) Flexible circuitry for an origami micro flight vehicle folded into a 3-dimensional origami structure. Includes solar cell-powered electromagnetic actuator with pressure and temperature sensors for altitude estimation. Reproduced with permission from [145].

Common strategies for internal integration mainly include the combination of liquid materials (e.g., liquid metals, ionic liquids, etc.) or solid materials (e.g., stretchable optical waveguides, CNTs, etc.). Liquid metals with both metallic and fluidic properties have a rich machine response behavior, which gives rise to their promising applications in sensor integration. For example, Robertson et al. [138] demonstrated the integration of a flexible, stretchable, liquid metal Ga-based strain sensor with a vacuum-driven soft body actuator. Thanks to the high compliance and deformability of liquid metal sensors, their unique SPA can generate characteristic deformations such as flexure and folding, which is one of the typical works based on the integration of pneumatic actuators with conductive fluids. A smart flexible pneumatic actuator, which applies stretchable electronics based on nano-Fe Ga In amalgam, as shown in Fig. 7A, was developed by Guo et al. [139] to realize applications in flexible manipulators and biomedicine. Besides, Liu et al. present a bimodal self-powered flexible sensor based on friction nanogenerators and giant magnetoelastic effect. The sensor has a magnetoelastic conductive film and an encapsulated liquid metal coil. The sensor was seamlessly integrated into pneumatic soft fingers (Fig. 7B) to realize the intelligent actuation sensing integration for soft robots [140]. Helps et al. propose a solution using a conductive working fluid, sodium chloride, to achieve a flexible fluid actuator with proprioceptive sensing. The working sodium chloride fluid drives the deformation of the brake and also acts as a strain-sensing element to detect the deformation of the actuator. This integrated sensing approach has advantages over conventional methods as it markedly reduces the size, mass, and complexity of the actuated sensing system [141]. This work is based on an ionic liquid-driven strategy for proprioceptive and bending motion, providing a completely new dimension for subsequent research. The integration of conductive liquids in the actuator–sensor fusion of soft robots demonstrates potential advantages. However, internal integration still faces challenges. Current limitations include difficulties in precise control, liquid durability, and compatibility with other materials. Future research could focus on optimizing the fine manipulation of conductive liquids, enhancing their durability, and exploring broader material compatibilities to achieve more comprehensive applications in the drive-sensor fusion of soft robots.

In addition to the integration of conductive and ionic liquids, there is a growing trend in applying emerging strategies for the internal integration of solid material to achieve actuation-sensing integration of soft robots. Optical waveguide-based photoelectric strain sensors are easy to fabricate, are chemically inert, and have low hysteresis and highly accurate output signals. As shown in Fig. 7C, Zhao et al. devised a strategy for the application of stretchable optical waveguides for solid-state sensing in prostheses. The authors created a flexible finger capable of achieving multi-modal movements such as multi-bending and elongation, curvature, elongation, and pressure detection, which was used to achieve external object sensing [142]. In addition, Dang et al. proposed a CNT-based internal strain sensor by integrating stretchable strain sensors in flexible mechanical fingers (Fig. 7D). They used a constant alternating current signal to detect the body deformation by monitoring the resistance of CNTs [143]. While solid material integration for actuation sensing integration can provide high sensing accuracy, these rigid additives can also affect the soft robot’s flexibility and a balance between the required conductivity and flexibility needs to be considered.

In addition to the above strategies, there has also been a lot of brand-new work recently on actuation sensing integration through internal integration of sensors. Sun et al. report a shape deformation scheme, which is accomplished by integrating a twist and curl actuator (TCA) and a customized SMP. Both actuation and sensing are realized through the TCA [144]. Its resistance varies with displacement. Based on this, they designed a new soft robot that can realize multi-modal land motion, as shown in Fig. 7E. In addition, Johnson et al. [145] designed a low-power electromagnetic actuator powered by a solar cell that allows a robot to generate a maximum actuation force of up to 200 mN in 25 ms. The researchers also fabricated a circuit on a folded origami structure that included a programmable microcontroller, a Bluetooth radio, solar energy harvesting circuitry, pressure sensors for estimating height, and temperature sensors (Fig. 7F). With this novel strategy, the internal integration of actuation sensing was realized.

Compared to traditional soft robots with independent actuation or independent sensing, the internal integration of drive sensing makes the robot more intelligent. In general, with the continuous breakthroughs in 4D printing, soft materials, and soft sensing technology, related technical problems will be able to be solved one by one in the future. Simultaneously, the internal integration of sensors must consider issues of sensitivity and precision. Further research is needed to ensure that the integration does not compromise the deformability and movement of the robot while maintaining sensing efficiency.

### Closed-loop systems based on the sensor feedback

In the preceding sections, we introduced 2 common approaches to the integration of flexible sensors in soft robots. While the incorporation of these sensors grants the robot sensing capabilities, the signals obtained from the sensors must undergo further processing to achieve more accurate actuation and control [146]. In this regard, creating a closed-loop system based on the sensor feedback signals is crucial in addressing this challenge. Through seamlessly integrating the sensor feedback with the actuation process, the soft robot gains unrivaled responsiveness, evolving into a sophisticated entity capable of real-time adaptation to environmental changes.

Traditional soft robots primarily utilize open-loop control, where the primary objective is to induce deformation or apply a fixed force to a specific position [28]. However, in applications such as intelligent rehabilitation equipment for biomedicine, soft robotic arms, and the detection of complex environments, monitoring the robot’s position and attitude is often essential. Since these data are susceptible to external disturbances, establishing a suitable closed-loop system becomes necessary [13]. Creating a closed-loop system requires the careful selection of appropriate system inputs and outputs based on the chosen sensing strategy. Typically, the transition to inductive currents based on sensor fundamentals such as pressure or curvature serves as a viable option. Subsequently, an appropriate modeling method must be selected, with 3D finite element analysis or 2D segmented constant curvature models being common choices. The feedback branch usually involves consideration of PID control, various signal processing methods, and the integration of sophisticated numerical analysis tools and kinetic modeling of the actuator.

**Fig. 8.**
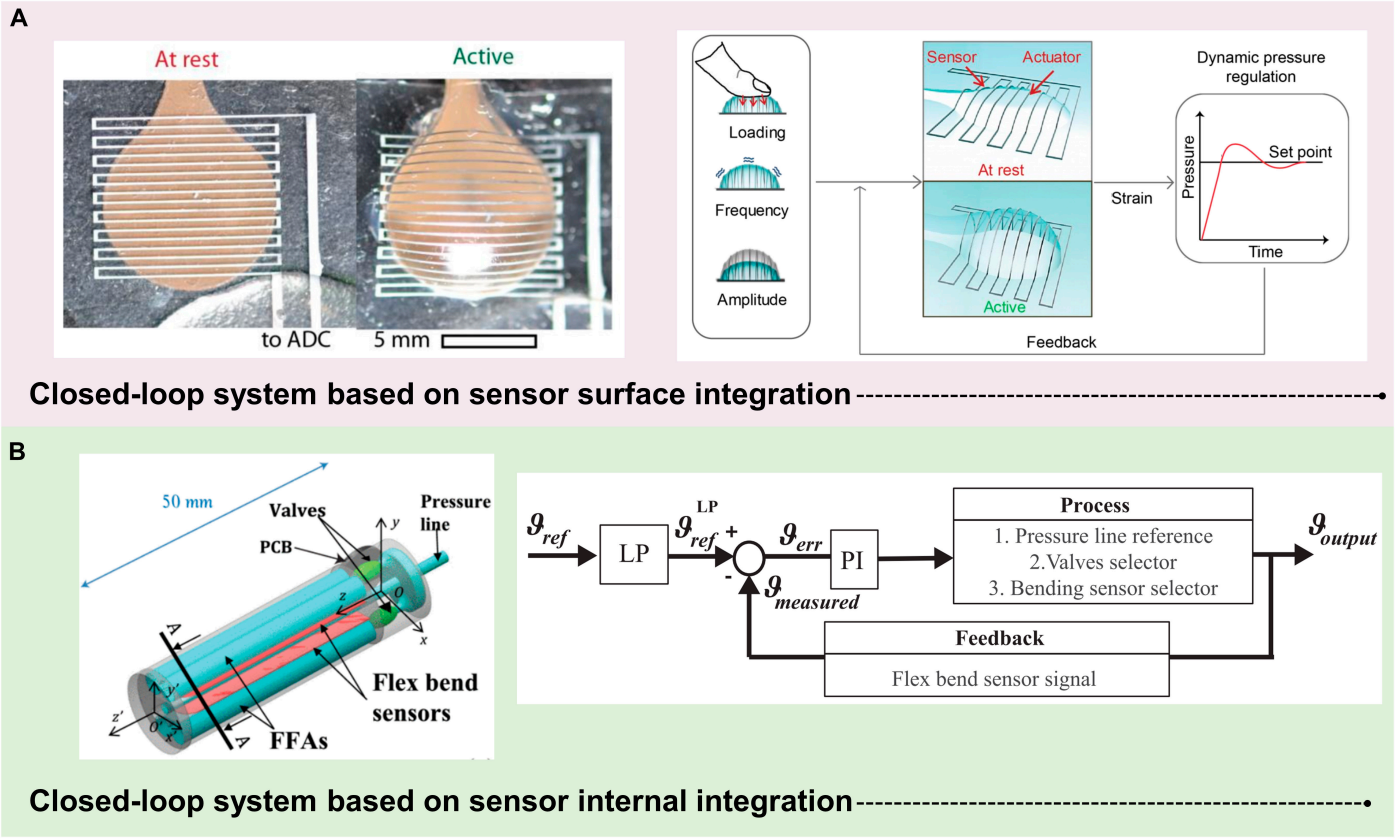
Closed-loop systems based on the sensor feedback. (A) Skin before and after inflation (left) and the corresponding closed loop (right). Reproduced with permission from [147]. (B) The integration of the bending sensor inside the actuator (left) and the closed-loop control loop (right), where LP stands for the low-pass filter and PI stands for the proportional integration controller. Reproduced with permission from [148].

For instance, Sonar et al. implemented closed-loop control of haptic feedback by designing a flexible skin for use with SPAs. The skin incorporates stretchable liquid metal strain sensors, as shown in Fig. 8A, showcasing the skin’s attitude before and after inflation and the corresponding closed-loop system [147]. When an external force is applied, the user needs to set the amplitude and frequency as inputs. Sensor-integrated actuators control the expansion of the actuator and the applied magnitude through active closed-loop feedback control, utilizing strain as an input to the feedback loop. For systems with internally integrated sensors, Gerboni et al. designed a feedback control using a low-pass filter (LP) and a proportional-integral controller (PI) for evaluating the dynamic response and positional accuracy of the integrated module [3,148]. A bending sensor was added inside the pressure-driven actuator, as shown in the left panel of Fig. 8B, and the closed-loop control loop is demonstrated in the right panel. The linear controller calculates the pressure received by the actuator, and the bend sensor signal is used as negative feedback to control the bending angle in the closed loop. Simultaneously, the calculated value of the pressure is provided to the pressure regulator of the main pressure line as a reference and the actuator is connected to the controlled pressure line by selecting the appropriate valve. The PI controller ensures zero position error under steady-state conditions, while the LP tunes the actuator dynamics. In addition, Felt et al. [149] designed a new smart braided sensor based on the change in inductance due to the actuator’s contraction. The closed-loop system uses an inductive value to estimate the tip angle of the manipulator, achieving closed-loop control by comparing the estimated value to a reference value. Similar designs include soft-touch gloves, relying on soft-pneumatic actuators that rely on sensory feedback signals to determine the success of a grip and recognize the shape of the grasped object [150].

Soft robots still encounter considerable challenges in practical closed-loop motion control [3]. One notable challenge arises from the susceptibility of sensing signals to environmental influences, making it challenging to implement rotary or linear encoders as commonly done in traditional rigid robots. Additionally, there is a need for smarter methods to be developed for dynamic mapping from drive space to task space. Machine learning emerges as a promising solution to address this complexity. In conclusion, these complex integrations not only enhance the operational functionality of robots but also lay the foundation for applications in various fields. They prove invaluable in medical wearable gear responding to physiological changes and facilitate breakthroughs in detection within extreme environments, enabling robots to navigate complex terrains with unparalleled precision.

### Exploration of possible strategies for integrated actuation and sensing

The complex actuation of soft robots often requires the application and integration of multiple actuation strategies to achieve their intelligent development. The integration of actuators in multiple locations throughout the body of a soft robot is often necessary to enable complex actuation modes [87]. When considering the interaction and perception of soft robots with the external environment, it is necessary to take into account whether they can achieve proprioceptive sensing (recording information related to their strain, bending, pressure, and torsion) and tactile sensing (perceiving external objects and external signals). Additionally, various strategies need to be considered for sensor wiring, power supply, data communication, and processing. However, the prevailing integrated actuation and sensing strategies based on e-skin and internal sensor integration often rely solely on pneumatic actuation as the driving method, which limits the ability of intelligent soft robots to exhibit multimodal motion. Therefore, it is crucial to explore integrated actuation and sensing methods under alternative driving strategies, identify their characteristics, and investigate whether there are approaches that can simultaneously integrate multimodal actuation and sensing in the integrated system.

**Fig. 9.**
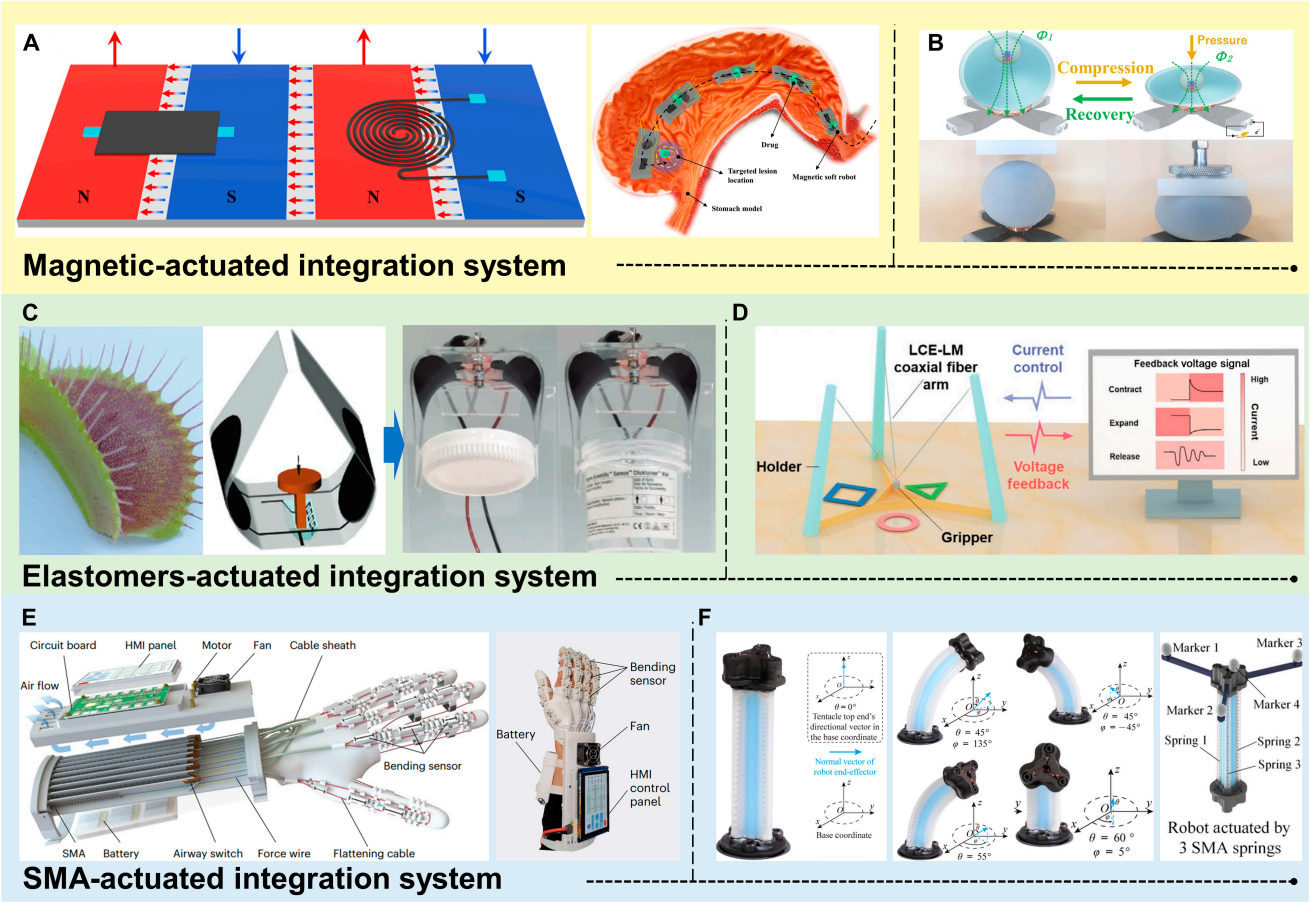
Intelligent robots with integrated sensing in multiple actuation modes. (A) Magnetic soft robot with integrated temperature and tactile sensing. Reproduced with permission from [151]. (B) An externally magnetically guided soft robot with the ability to sense its surroundings consisting of a magnetoelectric sensor and an actuation base. Reproduced with permission from [152]. (C) Flexible gripper with integrated dielectric elastomer and flexible resistive sensors. Reproduced with permission from [153]. (D) Soft 3-arm Delta robot based on LCE-LM coaxial fibers with sensing and actuation. Reproduced with permission from [154]. (E) A soft-packaged and portable rehabilitation glove driven by SMA actuators. Reproduced with permission from [155]. (F) Flexible soft robot tentacles with an integrated motion capture system actuated by 3 SMA springs. Reproduced with permission from [156].

Sensing integration for magnetically driven soft robots often requires consideration of whether the applied magnetic field interferes with the sensor performance. Additionally, selecting an appropriate communication method between the sensor and the robot control system is crucial to prevent interference with the robot’s motion. For example, Wang et al. employed a multi-material direct ink writing process to achieve the fabrication and integration of a magnetic soft robot with flexible sensing materials, as shown in Fig. 9A. The robot was composed of a magnetic body for magnetic actuation, a resistance-based sensor for temperature sensing, and a capacitance-based sensor for tactile sensing. Finally, targeted drug delivery experiments were conducted with the robot, which confirmed its excellent mobility and positioning capabilities [151]. The substantial advantages of magnetically driven soft robots are miniaturization and unfettered, and sensor integration needs to be carefully considered. Additionally, Yue et al. have produced an externally magnetically guided soft robot with the ability to sense its surroundings, as shown in Fig. 2A. The robot consists of a magnetoelectric sensor and a drive base. The base is driven by an external magnetic field and bent forward and backward in sequence to achieve advancement while integrating the soft body sense of the robot to sense the outside world to avoid obstacles and advance [152]. In addition to magnetically driven integrated systems, some studies employ elastomers for propulsion. In the realm of DE-driven robotics, the integration of sensors mandates meticulous contemplation of choices under high-voltage conditions. Simultaneously, it is imperative to deliberate upon the harmonious adaptation of sensing materials with the actuation framework. Luca et al. presented an example of intelligent soft robotic manipulation and multimodal sensing achieved through the use of DE actuators. As shown in Fig. 9C, they developed a gripper composed of DE-based actuators and a sensor network. It was capable of automatically detecting when an object was placed in the gripper and autonomously grasping the object [153]. This work is a reference for the study of autonomous soft-body devices, robots with distributed logic interacting with their environment. LCEs, owing to their exceptional orientational characteristics and piezoelectric properties in mechanical force fields, stand out as a commendable choice for achieving integrated actuation and sensing. In another example, Wei et al. prepared LCE fibers with a hollow structure and filled the fiber core with liquid metal (LM) to construct an LCE-LM coaxial fiber with sensing and driving functions. Researchers have built a soft 3-armed Delta robot, shown in Fig. 9D, which is competent for the task of object recognition and sorting within the range [154]. Besides the previously mentioned integrated systems, researchers have also explored systems driven by SMA. For example, Sui et al. presented a portable rehabilitation glove tailored for closed-loop FMSs (Fig. 9E). Featuring a touchable human–machine interface panel, a control system, and biomimetic finger stalls with bending sensors, the glove enables user input for FMS rehabilitation and provides real-time finger status display. The control system, driven by SMA actuators, employs pulse-width-modulation waves to govern finger movement. Validated for post-stroke individuals with hand impairments, the glove assists in both single-mode and switch-mode FMSs [155]. In addition, An et al. designed a 3-piece SMA spring-driven elastic soft robot tentacle, as shown in Fig. 9F. Efficient motion tracking of predefined trajectories and random trajectories was achieved by coordinating the bending and oscillating motions through the controller [156].

Common actuation strategies based on the integration of actuation sensing at this stage are pneumatic strategies based on pressure actuation strategies. In this section, we can also see examples based on electrical actuation, external response actuation, external force actuation, and shape memory actuation, but the amount of related work is limited and further related research is needed. The actuator, as a carrier of sensors, is key to the integration of actuation and sensing in flexible robots. Integration strategies based on pneumatic actuation make it easier to integrate flexible e-skins, and actuation-sensing integration strategies based on internal sensing integration make other actuation methods possible. The advent of conductive liquids and ionic liquids provides an effective way to integrate sensors internally for soft robots based on pneumatic drive strategies, and we can also consider flexible e-skins in SMAs, magnetic actuation techniques, and electric actuation techniques. This requires overcoming the drawbacks of the actuators themselves such as the high voltages of DEs, the magnetic requirements of magnetic actuation, and the temperature requirements of SMAs, to name but a few, as well as addressing the effects of easily repeated physical contact due to the thin structure of the flexible skin. This will require even further research to improve the compatibility of electrical, magnetic, and SMMs with flexible e-skins. With the continuous breakthrough of soft robot modeling methods and dynamic simulation techniques, complex integration strategies become possible; with the development of new flexible materials and additive manufacturing technologies, it will help improve actuator performance and sensor sensitivity to adapt to a wider range of application scenarios. Multimodal integration is another key point, and by integrating multiple actuators and sensors, soft robots can better perceive their environment and improve drive accuracy. With the continuous development of actuation and sensing technology, highly integrated intelligent soft robots will show their irreplaceable role in aerospace, extreme environment detection, and other fields.

## Summary and Outlook

**Fig. 10.**
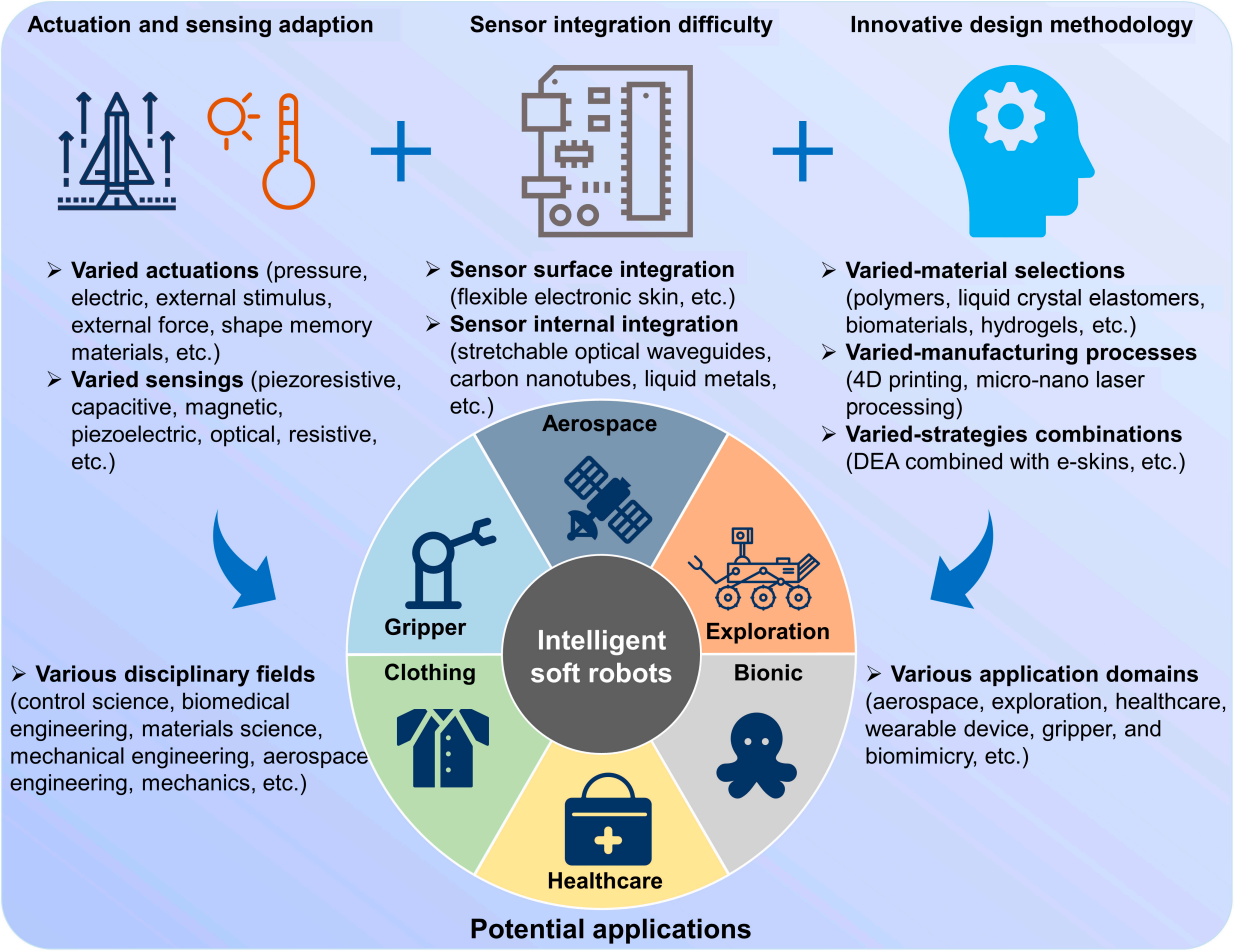
Remaining challenges and outlook on the integration of actuation and sensing in soft robots.

Soft robotics has experienced remarkable progress in recent years driven by continuous advancements in flexible actuation and sensing technology. These developments have given rise to increasingly intelligent soft robots. This review offers a thorough examination of diverse actuation and sensing strategies, elucidating the respective advantages and disadvantages associated with each. We also emphasize the significance of integrating actuation and sensing in soft robotics, presenting 2 primary integration methodologies, namely, flexible e-skin for sensor surface integration and internal integration of sensors. While substantial achievements have been made, ongoing efforts are imperative for the integration of actuators and sensors. The key to achieving intelligence in soft robotics lies in balancing the demands of actuator drive and sensor integration. It is crucial to select appropriate actuation and sensing strategies, considering specific application scenarios, such as navigating through small pipes or requiring substantial driving force. The successful integration of these strategies is important to realize the desired functionalities. Figure 10 provides an overview encompassing various obstacles, challenges, and future research directions, taking into account multiple influencing factors.

Firstly, when contemplating the performance compatibility of actuators and sensors, a thorough assessment of the working environment and motion requirements of the target robot becomes imperative. The judicious selection of suitable actuators, such as pressure-driven, electrical, externally stimulated, or those utilizing SMMs, is essential. Simultaneously, careful consideration must be given to the specific demands placed on the external environment by the target robot when choosing appropriate sensors, such as pressure resistance, electrical resistance, magnetic sensing, piezoelectric, optical, and capacitive sensors. Ensuring the performance compatibility of the chosen actuators and sensors is essential to guarantee both the efficiency of the actuators and the sensitivity of the sensors.

Secondly, within the current mainstream approaches, concerted efforts are underway to alleviate the integration challenges associated with embedding sensors on the surface of actuators. Present surface integration strategies for sensors primarily involve the use of flexible e-skins, which primarily facilitate the integration of inductive and capacitive sensors on soft material surfaces. However, it is noteworthy that research on surface integration for other sensing strategies remains an area ripe for exploration. In addition, internal integration strategies for sensors, the use of liquid metals, CNTs, and stretchable optical waveguides have shown promise. However, limitations related to working environment, conditions, and materials confine internal integration strategies predominantly to pressure-driven systems, with limited applicability to soft robots using other drive strategies. Exploring diverse internal sensor integration approaches based on different drive modes holds important potential for advancing intelligent applications in soft robots.

Thirdly, exploring entirely new design paradigms and innovative drive–sensor integration strategies is encouraged. The integration of more types of flexible materials, such as hydrogels, LCEs, and biocompatible polymers, into the main body of soft robots facilitates surface or internal sensor integration. Furthermore, the utilization of advanced manufacturing techniques like nano 3D/4D printing to construct more intricate structures on the surface of soft robots facilitates smoother integration of actuators and sensors [157–159]. It is essential to comprehensively acknowledge the advantages and drawbacks of various drive and sensor strategies. Further designs aspiring for intelligent robots should embrace a diverse array of drive and sensor strategy combinations, such as DE-driven actuators integrated with liquid metal sensors.

Fourthly, it is evident that soft robots can play an important role in various application domains through concrete solutions, necessitating the amalgamation of knowledge from control science, biomedical engineering, materials science, mechanical engineering, aerospace engineering, mechanics, and other multidisciplinary fields. Despite achieving control over numerous design variables in the realms of flexible actuation, flexible sensing, and actuator–sensor integration, it is pivotal to incorporate these design variables into a systematic framework. This approach aims to establish intelligent soft robots tailored to specific functionalities and scenarios. In the future, intelligent soft robots will explore novel applications in fields such as aerospace, exploration, healthcare, wearable devices, grippers, and biomimicry.

The emergence of new materials opens up new opportunities for designing and fabricating soft robots. The combination of rarely explored actuation and sensing integration strategies (e.g., DE integrated flexible e-skin, etc.) and the introduction of new materials will further enable the innovation of intelligent soft robots. It is expected that researchers experienced in interdisciplinary fields will find value in this paper, gaining an understanding of the fundamental actuation and sensing approaches for soft robots and considering how to achieve better integration of actuation and sensing, leading to breakthrough innovations in the field of soft robotics.

## Data Availability

The original data supporting this review are from previously reported studies and datasets, which have been cited. The processed data are available from the corresponding author upon request.

## References

[1] Kim S, Laschi C, Trimmer B. Soft robotics: A bioinspired evolution in robotics. Trends Biotechnol. 2013;31(5):287–294. 23582470 10.1016/j.tibtech.2013.03.002

[2] Hartmann F, Baumgartner M, Kaltenbrunner M. Becoming sustainable, the new frontier in soft robotics. Adv Mater. 2021;33(19):e2004413. 33336520 10.1002/adma.202004413 PMC11468029

[3] Trivedi D, Rahn CD, Kier WM, Walker ID. Soft robotics: Biological inspiration, state of the art, and future research. Appl Bionics Biomech. 2008;5(3):99–117.

[4] Wang Q, Yang S, Zhang L. Untethered micro/nanorobots for remote sensing: Toward intelligent platform. Nanomicro Lett. 2024;16(1):40. 10.1007/s40820-023-01261-9 PMC10689342 38032461

[5] Rus D, Tolley MT. Design, fabrication and control of soft robots. Nature. 2015;521(7553):467–475. 26017446 10.1038/nature14543

[6] Gul JZ, Sajid M, Rehman MM, Siddiqui GU, Shah I, Kim KH, Lee JW, Choi KH. 3D printing for soft robotics—A review. Sci Technol Adv Mater. 2018;19(1):243–262. 29707065 10.1080/14686996.2018.1431862 PMC5917433

[7] Stano G, Percoco G. Additive manufacturing aimed to soft robots fabrication: A review. Extreme Mech. Lett. 2021;42: Article 101079.

[8] Soleimanzadeh H, Rolfe B, Bodaghi M, Jamalabadi M, Zhang X, Zolfagharian A. Sustainable robots 4D printing. Adv Sustain Syst. 2023;7(12):2300289.

[9] Hann SY, Cui H, Nowicki M, Zhang LG. 4D printing soft robotics for biomedical applications. Addit Manuf. 2020;36: Article 101567.

[10] Wang D, Wang J, Shen Z, Jiang C, Zou J, Dong L, Fang NX, Gu G. Soft actuators and robots enabled by additive manufacturing. Annu Rev Control Robot Auton Syst. 2023;6:31–63.

[11] El-Atab N, Mishra RB, Al-Modaf F, Joharji L, Alsharif AA, Alamoudi H, Diaz M, Qaiser N, Hussain MM. Soft actuators for soft robotic applications: A review. Adv Intell Syst. 2020;2(10):2000128.

[12] Zaidi S, Maselli M, Laschi C, Cianchetti M. Actuation technologies for soft robot grippers and manipulators: A review. Curr Robot Rep. 2021;2(3):355–369.

[13] Pal A, Restrepo V, Goswami D, Martinez RV. Exploiting mechanical instabilities in soft robotics: Control, sensing, and actuation. Adv Mater. 2021;33(19):2006939. 10.1002/adma.202006939 33792085

[14] Schmitt F, Piccin O, Barbé L, Bayle B. Soft robots manufacturing: A review. Front Robot AI. 2018;5:84. 33500963 10.3389/frobt.2018.00084 PMC7805834

[15] Kim D, Kim SH, Kim T, Kang BB, Lee M, Park W, Ku S, Kim DW, Kwon J, Lee H, et al. Review of machine learning methods in soft robotics. PLoS One. 2021;16(2): Article e0246102. 33600496 10.1371/journal.pone.0246102 PMC7891779

[16] Das A, Nabi M. A review on soft robotics: Modeling, control and applications in human-robot interaction. Paper presented at: 2019 International Conference on Computing, Communication, and Intelligent Systems (ICCCIS). 2019 Oct 18–19, Greater Noida, India.

[17] Wang Y, Yang X, Chen Y, Wainwright DK, Kenaley CP, Gong Z, Liu Z, Liu H, Guan J, Wang T, et al. A biorobotic adhesive disc for underwater hitchhiking inspired by the remora suckerfish. Sci Robotics. 2017;2(10):eaan8072. 10.1126/scirobotics.aan8072 33157888

[18] Li G, Chen X, Zhou F, Liang Y, Xiao Y, Cao X, Zhang Z, Zhang M, Wu B, Yin S, et al. Self-powered soft robot in the Mariana trench. Nature. 2021;591(7848):66–71. 33658693 10.1038/s41586-020-03153-z

[19] Du Z, Fang H, Xu J. Snake-worm: A bi-modal locomotion robot. J Bionic Eng. 2022;19(5):1272–1287.

[20] Huang W, Xu Z, Xiao J, Hu W, Huang H, Zhou F. Multimodal soft robot for complex environments using bionic omnidirectional bending actuator. IEEE Access. 2020;8:193827–193844.

[21] Gu G, Zhang N, Xu H, Lin S, Yu Y, Chai G, Ge L, Yang H, Shao Q, Sheng X, et al. A soft neuroprosthetic hand providing simultaneous myoelectric control and tactile feedback. Nat Biomed Eng. 2023;7(4):589–598. 34400808 10.1038/s41551-021-00767-0

[22] Sui M, Ouyang Y, Jin H, Chai Z, Wei C, Li J, Xu M, Li W, Wang L, Zhang S. A soft-packaged and portable rehabilitation glove capable of closed-loop fine motor skills*. Nature* Nat Mach Intell. 2023;1149–1160.

[23] Li B, Cai Y, Jiang L, Liu L, Zhao Z, Chen G. A flexible morphing wing by soft wing skin actuation utilizing dielectric elastomer: Experiments and electro-aerodynamic model. Smart Mater Struct. 2020;29(1): Article 015031.

[24] Shi Q, Sun Z, le X, Xie J, Lee C. Soft robotic perception system with ultrasonic auto-positioning and multimodal sensory intelligence. ACS Nano. 2023;17(5):4985–4998. 36867760 10.1021/acsnano.2c12592

[25] Li M, Pal A, Aghakhani A, Pena-Francesch A, Sitti M. Soft actuators for real-world applications. Nat Rev Mater. 2022;7(3):235–249. 35474944 10.1038/s41578-021-00389-7 PMC7612659

[26] Wang H, Totaro M, Beccai L. Toward perceptive soft robots: Progress and challenges. Adv Sci. 2018;5(9):1800541. 10.1002/advs.201800541 PMC6145216 30250796

[27] Xiloyannis M, Alicea R, Georgarakis AM, Haufe FL, Wolf P, Masia L, Riener R. Soft robotic suits: State of the art, core technologies, and open challenges. IEEE Trans Robot. 2021;38(3):1343–1362.

[28] Polygerinos P, Correll N, Morin SA, Mosadegh B, Onal CD, Petersen K, Cianchetti M, Tolley MT, Shepherd RF. Soft robotics: Review of fluid-driven intrinsically soft devices; manufacturing, sensing, control, and applications in human-robot interaction. Adv Eng Mater. 2017;19(12):1700016.

[29] Gupta U, Qin L, Wang Y, Godaba H, Zhu J. Soft robots based on dielectric elastomer actuators: A review. Smart Mater Struct. 2019;28(10): Article 103002.

[30] Xia Y, He Y, Zhang F, Liu Y, Leng J. A review of shape memory polymers and composites: Mechanisms, materials, and applications. Adv Mater. 2021;33(6):2000713. 10.1002/adma.202000713 32969090

[31] Zou M, Li S, Hu X, Leng X, Wang R, Zhou X, Liu Z. Progresses in tensile, torsional, and multifunctional soft actuators. Adv Funct Mater. 2021;31(39):2007437.

[32] Georgopoulou A, Brancart J, Terryn S, Bosman AW, Norvez S, van Assche G, Iida F, Vanderborght B, Clemens F. Soft self-healing resistive-based sensors inspired by sensory transduction in biological systems. Appl Mater Today. 2022;29: Article 101638.

[33] Khan MA, Sun J, Li B, Przybysz A, Kosel J. Magnetic sensors—A review and recent technologies. Eng Res Express. 2021;3(2): Article 022005.

[34] Wang Q, Xiang N, Lang J, Wang B, Jin D, Zhang L. Reconfigurable liquid-bodied miniature machines: Magnetic control and microrobotic applications. Adv Intell Syst. 2023;6(2):2300108.

[35] Khatib M, Zohar O, Haick H. Self-healing soft sensors: From material design to implementation. Adv Mater. 2021;33(11):2004190. 10.1002/adma.202004190 33533124

[36] Chen D, Pei Q. Electronic muscles and skins: A review of soft sensors and actuators. Chem Rev. 2017;117(17):11239–11268. 28816043 10.1021/acs.chemrev.7b00019

[37] Jiang Y, Yin S, Dong J, Kaynak O. A review on soft sensors for monitoring, control, and optimization of industrial processes. IEEE Sensors J. 2021;21(11):12868–12881.

[38] Anwer AH, Khan N, Ansari MZ, Baek SS, Yi H, Kim S, Noh SM, Jeong C. Recent advances in touch sensors for flexible wearable devices. Sensors. 2022;22(12):4460. 35746242 10.3390/s22124460 PMC9229189

[39] Shih B, Shah D, Li J, Thuruthel TG, Park YL, Iida F, Bao Z, Kramer-Bottiglio R, Tolley MT. Electronic skins and machine learning for intelligent soft robots. Sci Robotics. 2020;5(41):eaaz9239. 10.1126/scirobotics.aaz9239 33022628

[40] Aubin CA, Gorissen B, Milana E, Buskohl PR, Lazarus N, Slipher GA, Keplinger C, Bongard J, Iida F, Lewis JA, et al. Towards enduring autonomous robots via embodied energy. Nature. 2022;602:393–402. 35173338 10.1038/s41586-021-04138-2

[41] Tan YJ, Susanto GJ, Ali HPA, Tee BCK. Progress and roadmap for intelligent self-healing materials in autonomous robotics. Adv Mater. 2020;33(19): Article e2002800. 33346389 10.1002/adma.202002800

[42] Keneth ES, Kamyshny A, Totaro M, Beccai L, Magdassi S. 3D printing materials for soft robotics. Adv Mater. 2020;33(19):2003387. 10.1002/adma.202003387 33164255

[43] Armanini C, Boyer F, Mathew AT, Duriez C, Renda F. Soft robots modeling: A structured overview. arXiv. 2022. https://arxiv.org/abs/2112.03645.

[44] Yasa O, Toshimitsu Y, Michelis MY, Jones LS, Filippi M, Buchner T, Katzschmann RK. An overview of soft robotics. Annu Rev Control Robot Auton Syst. 2023;6(1):1–29.

[45] Wang Q, Zhang J, Yu J, Lang J, Lyu Z, Chen Y, Zhang L. Untethered small-scale machines for microrobotic manipulation: From individual and multiple to collective machines. ACS Nano. 2023;17(14):13081–13109. 37440200 10.1021/acsnano.3c05328

[46] Klute GK, Czerniecki JM, Hannaford B. McKibben artificial muscles: Pneumatic actuators with biomechanical intelligence. Paper presented at: 1999 IEEE/ASME International Conference on Advanced Intelligent Mechatronics (Cat. No. 99TH8399); 1999 Sep 19–23; Atlanta, GA.

[47] De Volder M, Moers A, Reynaerts D. Fabrication and control of miniature McKibben actuators. Sensors Actuators A Phys. 2011;166(1):111–116.

[48] De Pascali C. 3D-printed biomimetic artificial muscles using soft actuators that contract and elongate. Sci Robotics. 2022;7(68):eabn4155. 10.1126/scirobotics.abn4155 35895921

[49] Zhang S, Zhang B, Zhao D, Gao Q, Wang ZL, Cheng T. Nondestructive dimension sorting by soft robotic grippers integrated with triboelectric sensor. ACS Nano. 2022;16(2):3008–3016. 35128922 10.1021/acsnano.1c10396

[50] Jones TJ, Jambon-Puillet E, Marthelot J, Brun PT. Bubble casting soft robotics. Nature. 2021;599(7884):229–233. 34759362 10.1038/s41586-021-04029-6

[51] Xavier MS, Fleming AJ, Yong YK. Finite element modeling of soft fluidic actuators: Overview and recent developments. Adv Intell Syst. 2021;3(2):2000187.

[52] Tang W, Zhong Y, Xu H, Qin K, Guo X, Hu Y, Zhu P, Qu Y, Yan D, Li Z, et al. Self-protection soft fluidic robots with rapid large-area self-healing capabilities. Nat Commun. 2023;14(1):6430. 37833280 10.1038/s41467-023-42214-5 PMC10576050

[53] Xie Q, Wang T, Yao S, Zhu Z, Tan N, Zhu S. Design and modeling of a hydraulic soft actuator with three degrees of freedom. Smart Mater Struct. 2020;29(12): Article 125017.

[54] Kurumaya S, Phillips BT, Becker KP, Rosen MH, Gruber DF, Galloway KC, Suzumori K, Wood RJ. A modular soft robotic wrist for underwater manipulation. Soft Robot. 2018;5(4):399–409. 29672216 10.1089/soro.2017.0097

[55] Zhao HC, Hussain AM, Duduta M, Vogt DM, Wood RJ, Clarke DR. Compact dielectric elastomer linear actuators. Adv Funct Mater. 2018;28(42):1804328.

[56] Jiang S, Tang C, Liu XJ, Zhao H, Tang C, Liu X-J, Zhao H. Long-life-cycle and damage-recovery artificial muscles via controllable and observable self-clearing process. Adv Eng Mater. 2022;24(4):2101017.

[57] Ji X, Liu X, Cacucciolo V, Imboden M, Civet Y, el Haitami A, Cantin S, Perriard Y, Shea H. An autonomous untethered fast soft robotic insect driven by low-voltage dielectric elastomer actuators. Sci Robotics. 2019;4(37):eaaz6451. 10.1126/scirobotics.aaz6451 33137720

[58] Chen Y, Zhao H, Mao J, Chirarattananon P, Helbling EF, Hyun NSP, Clarke DR, Wood RJ. Controlled flight of a microrobot powered by soft artificial muscles. Nature. 2019;575(7782):324–329. 31686057 10.1038/s41586-019-1737-7

[59] Rosset S, Shea HR. Flexible and stretchable electrodes for dielectric elastomer actuators. Appl Phys A. 2013;110(2):281–307.

[60] Tan MWM, Thangavel G, Lee PS. Enhancing dynamic actuation performance of dielectric elastomer actuators by tuning viscoelastic effects with polar crosslinking. NPG Asia Mater. 2019;11(1):62.

[61] He Q, Yin G, Vokoun D, Shen Q, Lu J, Liu X, Xu X, Yu M, Dai Z. Review on improvement, modeling, and application of ionic polymer metal composite artificial muscle. J Bionic Eng. 2022;19(2):279–298.

[62] Zhang X, Yu S, Li M, Zhang M, Zhang C, Wang M. Enhanced performance of IPMC actuator based on macroporous multilayer MCNTs/Nafion polymer. Sensors Actuators A Phys. 2022;339: Article 113489.

[63] Chung CK, Fung PK, Hong YZ, Ju MS, Lin CCK, Wu TC, Fung PK, Hong YZ, Ju MS, Lin CCK, et al. A novel fabrication of ionic polymer-metal composites (IPMC) actuator with silver nano-powders. Sensors Actuators B Chem. 2006;117(2):367–375.

[64] Yeom S-W, Oh I-K. A biomimetic jellyfish robot based on ionic polymer metal composite actuators. Smart Mater Struct. 2009;18(8): Article 085002.

[65] Hwang J, Wang WD. Shape memory alloy-based soft amphibious robot capable of seal-inspired locomotion. Adv Mater Technol. 2022;7(6):2101153.

[66] Mazzolai B, Margheri L, Cianchetti M, Dario P, Laschi C. Soft-robotic arm inspired by the octopus: II. From artificial requirements to innovative technological solutions. Bioinspir Biomim. 2012;7(2): Article 025005. 22617166 10.1088/1748-3182/7/2/025005

[67] Huang X, Kumar K, Jawed MK, Nasab AM, Ye Z, Shan W, Majidi C. Chasing biomimetic locomotion speeds: Creating untethered soft robots with shape memory alloy actuators. Sci Robotics. 2018;3(25):eaau7557. 10.1126/scirobotics.aau7557 33141693

[68] Ge Q, Sakhaei AH, Lee H, Dunn CK, Fang NX, Dunn ML. Multimaterial 4D printing with tailorable shape memory polymers. Sci Rep. 2016;6:31110. 27499417 10.1038/srep31110 PMC4976324

[69] Zhu Z, Cui C, Bai Y, Gao J, Jiang Y, Li B, Wang Y, Zhang Q, Qian W, Wei F, et al. Advances in precise structure control and assembly toward the carbon nanotube industry. Adv Funct Mater. 2022;32(11):2109401.

[70] Zhou P, Chen L, Yao L, Weng M, Zhang W. Humidity- and light-driven actuators based on carbon nanotube-coated paper and polymer composite. Nanoscale. 2018;10(18):8422–8427. 29637961 10.1039/c7nr09580e

[71] Gao Y-Y, Han B, Zhao WY, Ma ZC, Yu YS, Sun HB. Light-responsive actuators based on graphene. Front Chem. 2019;7:506. 31380350 10.3389/fchem.2019.00506 PMC6650529

[72] Deng H, Zhang C, Su JW, Xie Y, Zhang C, Lin J. Bioinspired multi-responsive soft actuators controlled by laser tailored graphene structures. J Mater Chem B. 2018;6(34):5415–5423. 32254600 10.1039/c8tb01285g

[73] White TJDJ, Broer DJ. Programmable and adaptive mechanics with liquid crystal polymer networks and elastomers. Nat Mater. 2015;14(11):1087–1098. 26490216 10.1038/nmat4433

[74] Yang Y, Wu Y, Li C, Yang X, Chen W, Wu Y, Li C, Yang X, Chen W. Flexible actuators for soft robotics. Adv Intell Syst. 2020;2(1):1900077.

[75] Yang M, Xu Y, Zhang X, Bisoyi HK, Xue P, Yang Y, Yang X, Valenzuela C, Chen Y, Wang L, et al. Bioinspired phototropic MXene-reinforced soft tubular actuators for omnidirectional light-tracking and adaptive photovoltaics. Adv Funct Mater. 2022;32(26):2201884.

[76] Kobayashi K, Yoon CK, Oh SH, Pagaduan JV, Gracias DH, Yoon C, Oh SH, Pagaduan JV, Gracias DH. Biodegradable thermomagnetically responsive soft untethered grippers. ACS Appl Mater Interfaces. 2019;11(1):151–159. 30525417 10.1021/acsami.8b15646

[77] Wang Q, Chan KF, Schweizer K, Du X, Jin D, Yu SCH, Nelson BJ, Zhang L. Ultrasound Doppler-guided real-time navigation of a magnetic microswarm for active endovascular delivery. Sci Adv. 2021;7(9):eabe5914. 33637532 10.1126/sciadv.abe5914 PMC7909881

[78] Ebrahimi N, Bi C, Cappelleri DJ, Ciuti G, Conn AT, Faivre D, Habibi N, Hošovský A, Iacovacci V, Khalil ISM, et al. Magnetic actuation methods in bio/soft robotics. Adv Funct Mater. 2021;31(11):2005137.

[79] Ze Q, Wu S, Nishikawa J, Dai J, Sun Y, Leanza S, Zemelka C, Novelino LS, Paulino GH, Zhao RR. Soft robotic origami crawler. Sci Adv. 2022;8(13):eabm7834. 35353556 10.1126/sciadv.abm7834 PMC8967224

[80] Dong Y, Wang L, Xia N, Yang Z, Zhang C, Pan C, Jin D, Zhang J, Majidi C, Zhang L. Untethered small-scale magnetic soft robot with programmable magnetization and integrated multifunctional modules. Sci Advances. 2022;8(25):eabn8932. 35731876 10.1126/sciadv.abn8932 PMC9217092

[81] Delph MA II, Fischer AS, Gauthier PW, Luna CHM, Clancy EA, Fischer GS. A soft robotic exomusculature glove with integrated sEMG sensing for hand rehabilitation. IEEE Int Conf Rehabil Robot. 2013;2013:6650426. 24187244 10.1109/ICORR.2013.6650426

[82] Kang BB, Choi H, Lee H, Cho KJ. Exo-glove poly II: A polymer-based soft wearable robot for the hand with a tendon-driven actuation system. Soft robotics. 2019;6(2):214–227. 30566026 10.1089/soro.2018.0006

[83] Chang E, Matloff LY, Stowers AK, Lentink D. Soft biohybrid morphing wings with feathers underactuated by wrist and finger motion. Sci Robot. 2020;5(38):eaay1246. 33022590 10.1126/scirobotics.aay1246

[84] Chen F, Xu W, Zhang H, Wang Y, Cao J, Wang MY, Ren H, Zhu J, Zhang YF. Topology optimized design, fabrication, and characterization of a soft cable-driven gripper. IEEE Robot Autom Lett. 2018;3(3):2463–2470.

[85] Rich SI, Wood RJ, Majidi C. Untethered soft robotics. Nat Electron. 2018;1(2):102–112.

[86] Hao Y, Zhang S, Fang B, Sun F, Liu H, Li H. A review of smart materials for the boost of soft actuators, soft sensors, and robotics applications. Chin J Mech Eng. 2022;35(1):1–16.

[87] Wehner M, Truby RL, Fitzgerald DJ, Mosadegh B, Whitesides GM, Lewis JA, Wood RJ. An integrated design and fabrication strategy for entirely soft, autonomous robots. Nature. 2016;536(7617):451–455. 27558065 10.1038/nature19100

[88] Katzschmann RK, Marchese AD, Rus D. Hydraulic autonomous soft robotic fish for 3D swimming. In: Hsieh MA, Khatib O, Kumar V, editorsExperimental Robotics: The 14th International Symposium on Experimental Robotics.Cham: Springer International Publishing; 2016. p. 405–420.

[89] Bartlett NW, Tolley MT, Overvelde JTB, Weaver JC, Mosadegh B, Bertoldi K, Whitesides GM, Wood RJ. A 3D-printed, functionally graded soft robot powered by combustion. Science. 2015;349(6244):161–165. 26160940 10.1126/science.aab0129

[90] Tang C, Du B, Jiang S, Shao Q, Dong X, Liu X-J, Zhao H. A pipeline inspection robot for navigating tubular environments in the sub-centimeter scale. Sci Robotics. 2022;7(66):eabm8597. 10.1126/scirobotics.abm8597 35613300

[91] Patel DK, Huang X, Luo Y, Mungekar M, Jawed MK, Yao L, Majidi C. Highly dynamic bistable soft actuator for reconfigurable multimodal soft robots. Adv Mater Technol. 2023;8(2):2201259.

[92] Zhong S, Xin Z, Hou Y, Li Y, Huang HW, Sun T, Shi Q, Wang H. Double-modal locomotion of a hydrogel ultra-soft magnetic miniature robot with switchable forms. Cyborg Bion Syst. 2024;5:0077. 10.34133/cbsystems.0077 PMC10907021 38435709

[93] Li W, Chen H, Yi Z, Fang F, Guo X, Wu Z, Gao Q, Shao L, Xu J, Meng G, et al. Self-vectoring electromagnetic soft robots with high operational dimensionality. Nat Commun. 2023;14(1):182. 36635282 10.1038/s41467-023-35848-y PMC9837125

[94] Sun B, Jia R, Yang H, Chen X, Tan K, Deng Q, Tang J. Magnetic arthropod millirobots fabricated by 3D-printed hydrogels. Adv Intell Syst. 2022;4(1):2100139.

[95] Li H, Li R, Wang K, Hu Y. Dual-responsive soft actuator based on aligned carbon nanotube composite/graphene bimorph for bioinspired applications. Macromol Mater Eng. 2021;306(8):2100166.

[96] Kastor N, Mukherjee R, Cohen E, Vikas V, Trimmer BA, White RD. Design and manufacturing of tendon-driven soft foam robots. Robotica. 2020;38(1):88–105.

[97] Schlagenhauf C, Bauer D, Chang K-H, King JP, Moro D, Coros S, Pollard N. Control of tendon-driven soft foam robot hands. Paper presented at: 2018 IEEE-RAS 18th International Conference on Humanoid Robots (Humanoids). 2018 Nov 6–9; Beijing, China.

[98] Zhu J, Pu MH, Chen H, Xu Y, Ding H, Wu ZG. Pneumatic and tendon actuation coupled multi-mode actuators for soft robots with broad force and speed range. SCIENCE CHINA Technol Sci. 2022;65(9):2156–2169.

[99] Umedachi T, Vikas V, Trimmer BA. Softworms: The design and control of non-pneumatic, 3D-printed, deformable robots. Bioinspir Biomim. 2016;11(2): Article 025001. 26963596 10.1088/1748-3190/11/2/025001

[100] Dong X, Luo X, Zhao H, Qiao C, Li J, Yi J, Yang L, Oropeza FJ, Hu TS, Xu Q, et al. Recent advances in biomimetic soft robotics: Fabrication approaches, driven strategies and applications. Soft Matter. 2022;18(40):7699–7734. 36205123 10.1039/d2sm01067d

[101] Jung J, Park M, Kim DW, Park YL. Optically sensorized elastomer air chamber for proprioceptive sensing of soft pneumatic actuators. IEEE Robot Autom Lett. 2020;5(2):2333–2340.

[102] Wang Y, Yue Y, Cheng F, Cheng Y, Ge B, Liu N, Gao Y. Ti3C2T x MXene-based flexible piezoresistive physical sensors. ACS Nano. 2022;16(2):1734–1758. 35148056 10.1021/acsnano.1c09925

[103] Chang H, Kim S, Jin S, Lee SW, Yang GT, Lee KY, Yi H. Ultrasensitive and highly stable resistive pressure sensors with biomaterial-incorporated interfacial layers for wearable health-monitoring and human-machine interfaces. ACS Appl Mater Interfaces. 2018;10(1):1067–1076. 29241330 10.1021/acsami.7b14048

[104] Zhang BX, Hou ZL, Yan W, Zhao QL, Zhan KT. Multi-dimensional flexible reduced graphene oxide/polymer sponges for multiple forms of strain sensors. Carbon. 2017;125:199–206.

[105] Alshawabkeh M, Alagi H, Navarro SE, Duriez C, Hein B, Zangl H, Faller LM. Highly stretchable additively manufactured capacitive proximity and tactile sensors for soft robotic systems. IEEE Trans Instrum Meas. 2023;72.

[106] Zhou X, Parida K, Halevi O, Liu Y, Xiong J, Magdassi S, Lee PS. All 3D-printed stretchable piezoelectric nanogenerator with non-protruding kirigami structure. Nano Energy. 2020;72: Article 104676.

[107] Wang XX, Song WZ, You MH, Zhang J, Yu M, Fan Z, Ramakrishna S, Long YZ. Bionic single-electrode electronic skin unit based on piezoelectric nanogenerator. ACS Nano. 2018;12(8):8588–8596. 30102853 10.1021/acsnano.8b04244

[108] Lepora NF. Soft biomimetic optical tactile sensing with the TacTip: A review. IEEE Sensors J. 2021;21(19):21131–21143.

[109] Galloway KC, Chen Y, Templeton E, Rife B, Godage IS, Barth EJ. Fiber optic shape sensing for soft robotics. Soft Robotics. 2019;6(5):671–684. 31241408 10.1089/soro.2018.0131 PMC6786339

[110] Kar D, George B, Sridharan K. A review on flexible sensors for soft robotics. In: *Systems for Printed Flexible Sensors: Design and Implementation*. Bristol (UK): IOP Publishing; 2022.

[111] Wang HB, de Boer G, Kow J, Alazmani A, Ghajari M, Hewson R, Culmer P. Design methodology for magnetic field-based soft tri-axis tactile sensors. Sensors. 2016;16 (9):1356. 27563908 10.3390/s16091356 PMC5038634

[112] Alfadhel A, Khan MA, Cardoso S, Leitao D, Kosel J. A magnetoresistive tactile sensor for harsh environment applications. Sensors. 2016;16(5):650. 27164113 10.3390/s16050650 PMC4883341

[113] Jin T, Sun ZD, Li L, Zhang Q, Zhu ML, Zhang ZX, Yuan GJ, Chen T, Tian YZ, Hou XY, et al. Triboelectric nanogenerator sensors for soft robotics aiming at digital twin applications. Nat Commun. 2020;11(1):5381. 33097696 10.1038/s41467-020-19059-3 PMC7585441

[114] Zhao J, Abbas A. A low-cost soft coiled sensor for soft robots. In: *ASME 2016* Dynamic Systems and Control Conference. Minneapolis (MN): American Society of Mechanical Engineers; 2016.

[115] Giordano G, Carlotti M, Mazzolai B. A perspective on cephalopods mimicry and bioinspired technologies toward proprioceptive autonomous soft robots. Adv Mater Technol. 2021;6(12):2100437.

[116] Preechayasomboon P, Rombokas E. Sensuator: A hybrid sensor-actuator approach to soft robotic proprioception using recurrent neural networks. Actuators. 2021;10(2):30.

[117] She Y, Liu SQ, Yu P, Adelson E. Exoskeleton-covered soft finger with vision-based proprioception and tactile sensing. Paper presented at: 2020 IEEE International Conference on Robotics and Automation (ICRA). 2020 May 31–Aug 31; Paris, France.

[118] Scimeca L, Hughes J, Maiolino P, Iida F. Model-free soft-structure reconstruction for proprioception using tactile arrays. IEEE Robot Autom Lett. 2019;4(3):2484.

[119] Shorthose O, Albini A, He L, Maiolino P. Design of a 3D-printed soft robotic hand with integrated distributed tactile sensing. IEEE Robot Autom Lett. 2022;7(2):3945–3952.

[120] Georgopoulou A, Hamelryckx S, Junge K, Eckey LM, Rogler S, Katzschmann R, Hughes J, Clemens F. A multi-material robotic finger with integrated proprioceptive and tactile capabilities produced with a circular process. Paper presented at: 2023 IEEE International Conference on Soft Robotics (RoboSoft); 2023 Apr 3–7; Singapore, Singapore.

[121] Cheng C, Yan Y, Guan M, Zhang J, Wang Y. Tactile sensing with a tendon-driven soft robotic finger. Paper presented at: 2021 9th International Conference on Control, Mechatronics and Automation (ICCMA); 2021 Nov 11–14; Belval, Luxembourg.

[122] Wang R, Wang S, Du S, Xiao E, Yuan W, Feng C. Real-time soft body 3D proprioception via deep vision-based sensing. IEEE Robot Autom Lett. 2020;5(2):3389.

[123] Homberg BS, Katzschmann RK, Dogar MR, Rus D. Haptic identification of objects using a modular soft robotic gripper. Paper presented at: 2015 IEEE/RSJ International Conference on Intelligent Robots and Systems (IROS); Sep 28; Hamburg, Germany.

[124] Zuo R, Zhou Z, Ying B, Liu X. A soft robotic gripper with anti-freezing ionic hydrogel-based sensors for learning-based object recognition. Paper presented at: 2021 IEEE International Conference on Robotics and Automation (ICRA); 2021 May 30; Xi’an, China.

[125] Ouyang W, He L, Albini A, Maiolino P. A modular soft robotic arm with embedded tactile sensors for proprioception. Paper presented at: 2022 IEEE 5th International Conference on Soft Robotics (RoboSoft); 2022 Apr 4–8; Edinburgh, United Kingdom.

[126] Soter G, Conn A, Hauser H, Rossiter J. Bodily aware soft robots: Integration of proprioceptive and exteroceptive sensors. Paper presented at: 2018 IEEE International Conference on Robotics and Automation (ICRA); 2018 May 21–25; Brisbane, QLD, Australia.

[127] Spielberg A, Amini A, Chin L, Matusik W, Rus D. Co-learning of task and sensor placement for soft robotics. IEEE Robot Autom Lett. 2021;6(2):1215.

[128] Chen J, Zhu Y, Chang X, Pan D, Song G, Guo Z, Naik N. Recent progress in essential functions of soft electronic skin. Adv Funct Mater. 2021;31(42):2104686.

[129] Levi A, Piovanelli M, Furlan S, Mazzolai B, Beccai L. Soft, transparent, electronic skin for distributed and multiple pressure sensing. Sensors. 2013;13(5):6578–6604. 23686140 10.3390/s130506578 PMC3690071

[130] Georgopoulou A, Bosman AW, Brancart J, Vanderborght B, Clemens F. Supramolecular self-healing sensor fiber composites for damage detection in piezoresistive electronic skin for soft robots. Polymers. 2021;13(17). 10.3390/polym13172983 PMC8433753 34503023

[131] Sengupta D, Romano J, Kottapalli AGP. Electrospun bundled carbon nanofibers for skin-inspired tactile sensing, proprioception and gesture tracking applications. Npj Flex Electron. 2021;5(1):29.

[132] Yu Y, Li JH, Solomon SA, Min JH, Tu JB, Guo W, Xu CH, Song Y, Gao W. All-printed soft human-machine interface for robotic physicochemical sensing. Sci Robot. 2022;7(67):eabn0495. 35648844 10.1126/scirobotics.abn0495 PMC9302713

[133] Si ZL, Yu TC, Morozov K, McCann J, Yuan WZ. RobotSweater: Scalable, generalizable, and customizable machine-knitted tactile skins for robots. Paper presented at: IEEE International Conference on Robotics and Automation (ICRA); 2023 May 29–Jun 2; London, United Kingdom.

[134] Wang W, Jiang Y, Zhong D, Zhang Z, Choudhury S, Lai JC, Gong H, Niu S, Yan X, Zheng Y, et al. Neuromorphic sensorimotor loop embodied by monolithically integrated, low-voltage, soft e-skin. Science. 2023;380(6646):735–742. 37200416 10.1126/science.ade0086

[135] Truby RL, Santina CD, Rus D. Distributed proprioception of 3D configuration in soft, sensorized robots via deep learning. IEEE Robot Autom Lett. 2020;5(2):3299–3306.

[136] Booth JW, Shah D, Case JC, White EL, Yuen MC, Cyr-Choiniere O, Kramer-Bottiglio R. OmniSkins: Robotic skins that turn inanimate objects into multifunctional robots. Sci Robotics. 2018;3(22):eaat1853. 10.1126/scirobotics.aat1853 33141754

[137] Kim T, Yoon SJ, Park YL. Soft inflatable sensing modules for safe and interactive robots. IEEE Robot Autom Lett. 2018;3(4):3216–3223.

[138] Robertson MA, Dejace L, Lacour SPS, Paik JK, Bi-modal control of vacuum-powered soft pneumatic actuators with embedded liquid metal-based strain sensitive skin. Paper presented at: 2019 2nd IEEE International Conference on Soft Robotics (RoboSoft); 2019 Apr 14–18; Seoul, South Korea.

[139] Guo R, Wang H, Duan M, Yu W, Wang X, Liu J. Stretchable electronics based on nano-Fe GaIn amalgams for smart flexible pneumatic actuator. Smart Mater Struct. 2018;27(8): Article 085022.

[140] Liu W, Duo Y, Chen X, Chen B, Bu T, Li L, Duan J, Zuo Z, Wang Y, Fang B, et al. An intelligent robotic system capable of sensing and describing objects based on bimodal, self-powered flexible sensors. Adv Funct Mater. 2023;33(41):2306368.

[141] Helps T, Rossiter J. Proprioceptive flexible fluidic actuators using conductive working fluids. Soft Robot. 2018;5(2):175–189. 29211627 10.1089/soro.2017.0012 PMC5905876

[142] Zhao H. Optoelectronically innervated soft prosthetic hand via stretchable optical waveguides. Sci Robot. 2016;1(1):eaai7529. 33157858 10.1126/scirobotics.aai7529

[143] Dang W, Hosseini ES, Dahiya R. Soft robotic finger with integrated stretchable strain sensor. Paper presented at: 2018 IEEE SENSORS. 2018 Oct 28–31; New Delhi, India.

[144] Sun J, Lerner E, Tighe B, Middlemist C, Zhao J. Embedded shape morphing for morphologically adaptive robots. Nat Commun. 2023;14(1):6023. 37758737 10.1038/s41467-023-41708-6 PMC10533550

[145] Johnson K, Arroyos V, Ferran A, Villanueva R, Yin D, Elberier T, Aliseda A, Fuller S, Iyer V, Gollakota S. Solar-powered shape-changing origami microfliers. Sci Robot. 2023;8(82):eadg4276. 37703382 10.1126/scirobotics.adg4276

[146] Cai M, Wang Q, Qi Z, Jin D, Wu X, Xu T, Zhang L. Deep reinforcement learning framework-based flow rate rejection control of soft magnetic miniature robots. In: *IEEE Transactions on Cybernetics*. IEEE; 2022. p. 7699–7711. 10.1109/TCYB.2022.3199213 36070281

[147] Sonar HA, Gerratt AP, Lacour SP, Paik J. Closed-loop haptic feedback control using a self-sensing soft pneumatic actuator skin. Soft Robot. 2020;7(1):22–29. 31549908 10.1089/soro.2019.0013

[148] Gerboni G, Diodato A, Ciuti G, Cianchetti M, Menciassi A. Feedback control of soft robot actuators via commercial flex bend sensors. IEEE/ASME Trans Mechatron. 2017;22(4):1881–1888.

[149] Felt W, Chin KY, Remy CD. Smart braid feedback for the closed-loop control of soft robotic systems. Soft Robot. 2017;4(3):261–273. 29062629 10.1089/soro.2016.0056 PMC5649447

[150] Low JH, Lee WW, Khin PM, Thakor NV, Kukreja SL, Ren HL, Yeow CH. Hybrid tele-manipulation system using a sensorized 3-D-printed soft robotic gripper and a soft fabric-based haptic glove. IEEE Robot Autom Lett. 2017;2(2):880–887.

[151] Wang Z, Wu Y, Zhu B, Chen Q, Wang L, Zhao Y, Sun D, Zheng J, Wu D. A magnetic soft robot with multimodal sensing capability by multimaterial direct ink writing. Addit Manuf. 2023;61: Article 103320.

[152] Yue Y, Wang Q, Wu Z, Huang J, Chen D, Ma Z, Su B. Buoy structural high-sensitive magnetoelectric sensors for soft robots’ tactile sensing and avoiding obstacles. Appl Mater Today. 2022;27: Article 101440.

[153] Ciarella L, Richter A, Henke EFM. Integrated logic for dielectric elastomers: Replicating the reflex of the venus flytrap. Adv Mater Technol. 2023;8(12):2202000.

[154] Liao W, Yang Z. The integration of sensing and actuating based on a simple design fiber actuator towards intelligent soft robots. Adv Mater Technol. 2022;7(6):2101260.

[155] Sui ML, Ouyang YM, Jin H, Chai ZY, Wei CY, Li JY, Xu M, Li WH, Wang L, Zhang SW. A soft-packaged and portable rehabilitation glove capable of closed-loop fine motor skills. Nat Mach Intell. 2023;5(10):1–12.

[156] An X, Cui Y, Sun H, Shao Q, Zhao H. Active-cooling-in-the-loop controller design and implementation for an SMA-driven soft robotic tentacle. IEEE Trans Robot. 2023;39(3):2325–2341.

[157] Lyu Z, Wang JL, Chen YF. 4D printing: Interdisciplinary integration of smart materials, structural design, and new functionality. Intl J Extrem Manuf. 2023;5(3):032011.

[158] Lyu Z, Koh JJ, Lim GJH, Zhang D, Xiong T, Zhang L, Liu S, Duan J, Ding J, Wang J, et al. Direct ink writing of programmable functional silicone-based composites for 4D printing applications. Interdiscip Mater. 2022;1(4):507–516.

[159] Li J, Li M, Koh JJ, Wang J, Lyu Z. 3D-printed biomimetic structures for energy and environmental applications. DeCarbon. 2023;3: Article 100026.

[160] Atalay O, Atalay A, Gafford J, Walsh C. A highly sensitive capacitive-based soft pressure sensor based on a conductive fabric and a microporous dielectric layer. Adv Mater Technol. 2018;3(1).

[161] Belforte G, Eula G, Ivanov A, Sirolli S. Soft pneumatic actuators for rehabilitation. Actuators. 2014;3(2):84–106.

[162] Al-Fahaam H, Davis S, Nefti-Meziani S. Power assistive and rehabilitation wearable robot based on pneumatic soft actuators. Paper presented at: 2016 21st International Conference on Methods and Models in Automation and Robotics (MMAR); 2016 Aug 29–Sep 1; Miedzyzdroje, Poland.

[163] Feng Y, Ide T, Nabae H, Endo G, Sakurai R, Ohno S, Suzumori K. Safety-enhanced control strategy of a power soft robot driven by hydraulic artificial muscles. ROBOMECH J. 2021;8(1):10.

[164] Franke M, Ehrenhofer A, Lahiri S, Henke E-FM, Wallmersperger T, Richter A. Dielectric elastomer actuator driven soft robotic structures with bioinspired skeletal and muscular reinforcement. Front Robot AI. 2020;7:510757. 33501298 10.3389/frobt.2020.510757 PMC7805688

[165] Luqman M, Shaikh HM, Anis A, al-Zahrani SM, Alam MA. A convenient and simple ionic polymer-metal composite (IPMC) actuator based on a platinum-coated sulfonated poly(ether ether ketone)–polyaniline composite membrane. Polymers. 2022;14(4):668. 35215581 10.3390/polym14040668 PMC8879763

[166] Ishiki A, Nabae H, Kodaira A, Suzumori K. PF-IPMC: Paper/fabric assisted IPMC actuators for 3D crafts. IEEE Robot Autom Lett. 2020;5(3):4035–4041.

[167] Jin H, Dong E, Xu M, Liu C, Alici G, Jie Y. Soft and smart modular structures actuated by shape memory alloy (SMA) wires as tentacles of soft robots. Smart Mater Struct. 2016;25(8): Article 085026.

[168] Hassan MMT. A bioinspired soft robotic gripper for adaptable and effective grasping. Soft Robotics. 2015;2(3):107–116.

[169] Xu F, Wang H, Wang J, Au KWS, Chen W. Underwater dynamic visual servoing for a soft robot arm with online distortion correction. IEEE/ASME Trans Mechatron. 2019;24(3):979–989.

[170] Calisti M, Arienti A, Renda F, Levy G, Hochner B, Mazzolai B, Dario P, Laschi C. Design and development of a soft robot with crawling and grasping capabilities. Paper presented at: 2012 IEEE International Conference on Robotics and Automation; 2012 May 14–18; Saint Paul, MN.

[171] Ramezani A, Chung SJ, Hutchinson S. A biomimetic robotic platform to study flight specializations of bats. Sci Robot. 2017;2(3):eaal2505. 33157861 10.1126/scirobotics.aal2505

